# Supplementary figures and images for: Classification of symptom subtypes in patients with multiple myeloma during treatment: a cross-sectional survey study in China
Source: BMJ Open. 2023 Mar 14;13(3):e066467. doi: 10.1136/bmjopen-2022-066467 (PMC10016277; doi:10.1136/bmjopen-2022-066467)

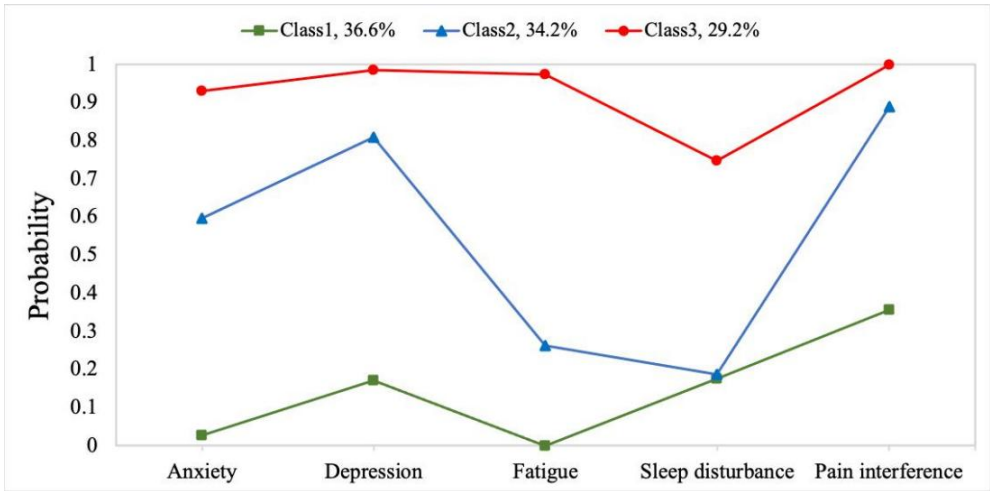

Supplementary Figure 1 The three-class latent profile analysis model

Supplement: Supplementary data [file bmjopen-2022-066467supp001.pdf]

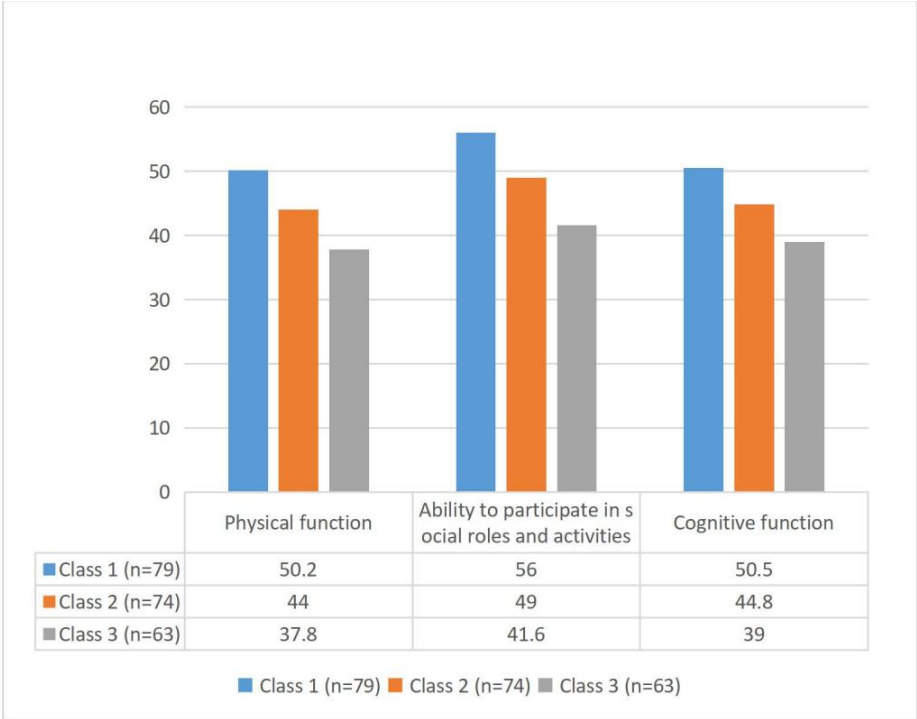

**Supplementary Figure 2** Functional status across the identified latent classes

Supplement: Supplementary data [file bmjopen-2022-066467supp002.pdf]
