# Supplementary material for: Classification of symptom subtypes in patients with multiple myeloma during treatment: a cross-sectional survey study in China
Source: BMJ Open. 2023 Mar 14;13(3):e066467. doi: 10.1136/bmjopen-2022-066467 (PMC10016277; doi:10.1136/bmjopen-2022-066467)
Supplement: Author's manuscript [file bmjopen-2022-066467.draft_revisions.pdf]

BMJ Open

BMJ Open is committed to open peer review. As part of this commitment we make the peer review history of every article we publish publicly available.

When an article is published we post the peer reviewers' comments and the authors' responses online. We also post the versions of the paper that were used during peer review. These are the versions that the peer review comments apply to.

The versions of the paper that follow are the versions that were submitted during the peer review process. They are not the versions of record or the final published versions. They should not be cited or distributed as the published version of this manuscript.

BMJ Open is an open access journal and the full, final, typeset and author-corrected version of record of the manuscript is available on our site with no access controls, subscription charges or pay-per-view fees (http://bmjopen.bmj.com).

If you have any questions on BMJ Open's open peer review process please email
info.bmjopen@bmj.com

BMJ Open

Subtypes of symptoms in patients with multiple myeloma during treatment: A latent class analysis approach

Journal:	*BMJ Open*
Manuscript ID	bmjopen-2022-066467
Article Type:	Original research
Date Submitted by the Author:	16-Jul-2022
Complete List of Authors:	Yu, Chunfang; The Second Affiliated Hospital of Guilin Medical University<br>Cai, Tingting; Fudan University School of Nursing<br>Zhou, Tingting; Fudan University School of Nursing<br>Zeng, Ning; Affiliated Hospital of Guilin Medical University<br>Liang, Xin; Guangxi Normal University<br>Pan, Guihua; Affiliated Hospital of Guilin Medical University<br>Ouyang, Wei; School of Nursing, Guilin Medical University<br>Yuan, Changrong; Fudan University School of Nursing,
Keywords:	Myeloma < HAEMATOLOGY, Adult oncology < ONCOLOGY, CHEMOTHERAPY, Lymphoma < HAEMATOLOGY

SCHOLARONE™ Manuscripts

BMJ

I, the Submitting Author has the right to grant and does grant on behalf of all authors of the Work (as defined in the below author licence), an exclusive licence and/or a non-exclusive licence for contributions from authors who are: i) UK Crown employees; ii) where BMJ has agreed a CC-BY licence shall apply, and/or iii) in accordance with the terms applicable for US Federal Government officers or employees acting as part of their official duties; on a worldwide, perpetual, irrevocable, royalty-free basis to BMJ Publishing Group Ltd ("BMJ") its licensees and where the relevant Journal is co-owned by BMJ to the co-owners of the Journal, to publish the Work in this journal and any other BMJ products and to exploit all rights, as set out in our licence.

The Submitting Author accepts and understands that any supply made under these terms is made by BMJ to the Submitting Author unless you are acting as an employee on behalf of your employer or a postgraduate student of an affiliated institution which is paying any applicable article publishing charge ("APC") for Open Access articles. Where the Submitting Author wishes to make the Work available on an Open Access basis (and intends to pay the relevant APC), the terms of reuse of such Open Access shall be governed by a Creative Commons licence – details of these licences and which Creative Commons licence will apply to this Work are set out in our licence referred to above.

Other than as permitted in any relevant BMJ Author's Self Archiving Policies, I confirm this Work has not been accepted for publication elsewhere, is not being considered for publication elsewhere and does not duplicate material already published. I confirm all authors consent to publication of this Work and authorise the granting of this licence.

For peer review only

0

Title Page

Manuscript category: research paper

Title: Subtypes of symptoms in patients with multiple myeloma during treatment: A latent class analysis approach

Short title: cancer-related symptoms profiles

Authors and Affiliation:

Authors： Chunfang Yu[1#], MD; Tingting Cai[2#], PhD; Tingting Zhou[2], MD; Ning Zeng[3], MD; Xin Liang[4], PhD; Guihua Pan[3], RN; Wei Ouyang[5], RN; Changrong Yuan[2*], PhD

Note: Chunfang Yu and Tingting Cai made equal contributions to this manuscript and should be regarded as joint first author.

Affiliations：

1 The Second Affiliated Hospital of Guilin Medical University, Guilin, China

2 School of Nursing, Fudan University, Shanghai, China

3 Affiliated Hospital of Guilin Medical University, Guilin, China

4 School of Mathematics and Statistics, Guangxi Normal University, Guilin, China

5 School of Nursing, Guilin Medical University, Guilin, China

Correspondence: Changrong Yuan, PhD, RN, FAAN, Fudan University, School of Nursing, 305 Fenglin Road Shanghai 200032, China (yuancr@fudan.edu.cn), Tel: 02164431803.

Funding

This work was supported by the Science and Technology Project of Guilin City (20210227-8-5) and Project of the Guangxi Education Department (2021KY0521).

Word counts: 3,569 words

Acknowledgements

For peer review only

We thank the study sites, which enabled us to recruit the patients required for this study.

Conflict of interest

None.

Abstract

Objectives: This study aimed to classify subgroups of cancer-related symptoms in patients with multiple myeloma (MM) during treatment and examine between-group differences in demographic and clinical characteristics in addition to functional status.

Methods: Using a convenience sampling method, questionnaires were distributed to patients with MM in two hospitals in Guilin, China. The patients were categorized into subgroups based on cancer-related symptoms using a latent class analysis. An analysis of covariance was performed to examine how demographic and clinical characteristics and functional status differed among the subgroups.

Results: A total of 216 patients completed the survey, with an average age of 60.3 years. A three-class solution was identified: low symptom group (Class 1, 36.6%); high anxiety, depression, and pain interference group (Class 2, 34.2%); and high symptom group (Class 3, 29.2%). Patients with low monthly family income (odds ratio [OR]=3.14, P=0.010) and complications of MM bone disease (OR=2.95, P=0.029) were more likely to belong to Class 2. The strongest predictors of high symptoms were taking painkillers, antidepressants, or hypnotic drugs (OR=3.68, P=0.012) and daily step counts less than 5000 (OR=2.52, P=0.039) in Class 3. Functional levels were negatively correlated with symptom burden, in which patients in Class 3 reported significantly higher functional levels and Class 1 scored the lowest (P<0.05).

For peer review only

Conclusion: Patients with MM experienced varying degrees of symptoms during treatment. The identification of patients with high symptom management needs might focus on assessing demographic and clinical characteristics, in addition to functional status.

Keywords: multiple myeloma, patient-reported outcomes, symptoms, function, latent class analysis

Strengths and limitations of this study

1. To our knowledge, this study is one of the few to classify subgroups of cancer-related symptoms in patients with multiple myeloma (MM) during treatment and examine between-group differences in demographic and clinical characteristics in addition to functional status，especially in the Chinese cancer population.

2. The results will facilitate nurses to identify patients with high symptom management requirements and should focus on assessing demographic and clinical characteristics and functional status, so as to design interventions for high-risk individuals.

3. The survey only included multiple myeloma patients in tertiary hospitals.

4. this was a cross-sectional study that failed to explore the symptoms of trajectories over time.

For peer review only

Subtypes of symptoms in patients with multiple myeloma during treatment: A latent class analysis approach

Chunfang Yu[1#], MD; Tingting Cai[2#], PhD; Tingting Zhou[2], MD; Ning Zeng[3], MD; Xin Liang[4], PhD; Guihua Pan[3], RN; Wei Ouyang[5], RN; Changrong Yuan[2*], PhD

Note: Chunfang Yu and Tingting Cai made equal contributions to this manuscript and should be regarded as joint first author.

[1]The Second Affiliated Hospital of Guilin Medical University, Guilin, China

[2]School of Nursing, Fudan University, Shanghai, China

[3]Affiliated Hospital of Guilin Medical University, Guilin, China

[4]School of Mathematics and Statistics, Guangxi Normal University, Guilin, China

[5]School of Nursing, Guilin Medical University, Guilin, China

For peer review only

INTRODUCTION

Multiple myeloma (MM) is the second most common hematologic cancer worldwide.[1] Although still incurable, advancements in the management of MM have extended survival and turned MM into a chronic disease. The median age at diagnosis of MM is 73 years.[1] Therefore, MM is more likely to be a cancer primarily of older age, with a median age at diagnosis of >70 years. During MM treatment, older patients have a higher rate of comorbid chronic and progressive diseases and require more care to deal with multiple treatment-related issues, including symptoms and functional limitations, than younger patients.[2] Patients with MM frequently report symptoms, including pain, fatigue, and depression, after diagnosis and throughout treatment.[3] With increasing survival rates, increased attention has been paid to symptom control in this population.

Although an increasing number of published studies have reported advancements in MM treatment, little is known about the symptoms of patients with MM during treatment.[4,5,6] Monitoring symptoms, as complementary to traditional laboratory tests, contributes to the identification of individuals who require supportive interventions. The effects of MM and its treatment may have an effect on patients' physical and emotional well-being, in addition to their social function. There is a paucity of data regarding the symptoms experienced by patients with MM, especially those undergoing therapy. Symptoms may affect cancer treatment options and outcomes. Individual symptom experiences must be understood to identify patients who are at risk for severe symptoms.

Evidence suggests that direct patient reports are the best way to capture information on patients' experiences in response to the disease and related treatment.[2,6,7] Patient-reported outcomes (PROs) are highly recommended to measure symptoms and treatment effectiveness in patients with MM.[7] Specifically, among patients undergoing therapy, PROs can provide a better understanding of the magnitude and temporal trends of symptoms from the perspective of individual patients. Additionally, using PROs to capture patients' symptoms is necessary to

For peer review only

perform value-based and patient-centered care and to enable more accurate symptom evaluation [7].

To date, the heterogeneity of symptoms has not been captured among patients with MM. Patients experience multiple symptoms that potentially interact with each other and that occur in clusters of clinically distinct subgroups. Statistical advancements in modeling contribute to the identification of subtypes and might be especially useful in distinguishing patients at risk, in which latent class analysis (LCA) is a person-oriented approach to classify individuals based on their symptom profiles and estimate distinct patterns.[8]

There is no evidence to explore the symptom profiles resulting from the disease and treatment in this population. Therefore, this study aimed to classify subgroups of patients with MM by incorporating PRO instruments and to examine between-group differences in demographic and clinical characteristics, in addition to functional status.

METHODS

Study design and sample

From July to December 2021, a cross-sectional survey was administered to a convenience sample of patients with MM in the Department of Hematology of a tertiary hospital in Guilin, China. Eligible patients were aged >18 years, diagnosed with primary MM, hospitalized for treatment, and were able to speak and write Mandarin Chinese. Patients with other cancers or mental illnesses were excluded from this study.

Study instruments

Demographic and clinical data

Demographic and socioeconomic data included age, sex, marital status, educational background, employment status, monthly family income, medical insurance, daily sleep duration, and daily step counts. The MM type, disease stage, drug usage, complications, treatments, and body mass

For peer review only

index (BMI) were retrieved from the medical records.

Patient-Reported Outcomes Measurement Information System (PROMIS)-57

Symptom experiences were measured using the Patient-Reported Outcomes Measurement Information System (PROMIS)-57 instrument. The PROMIS-57 consists of 57 items and includes five symptoms (anxiety, depression, fatigue, sleep disturbance, pain interference) and two functional domains (physical function, ability to participate in social roles and activities), along with one additional pain intensity item [9]. The instrument adopts a Likert scale (0-5) to assess the severity of symptoms or functional limitation, except for pain intensity, which has 11 response options (ranging from 0 to 10). The raw score of all outcome scores ranged from 4 to 20 and was converted into T-scores with a mean of 50 and a standard deviation (SD) of 10 in the scoring manual [10]. A higher score indicates poor functional status; however, for the symptom domain, this is reversed, where higher scores reflect a high symptom burden [9]. The Cronbach α coefficient ranged from 0.85 to 0.95 in this study.

PROMIS-Cognitive Function Short Form

The four-item PROMIS-Cognitive Function Short Form measures perceived cognitive function in the past 7 days. Each question was given a response of 1 ("never") to 5 ("very often").[11] The total raw score ranges from 4 to 20 and must be converted into T-scores. Similar to the PROMIS-57, lower T-scores indicate greater subjective cognitive difficulty. The Cronbach α for the scale was 0.93.

Data collection

The study protocol was approved by the Ethics Committee of the Second Affiliated Hospital of Guilin Medical University (No. GLKJJH010). Eligible patients were approached and provided with information about the study by assistant nurses. Written informed consent was obtained

For peer review only

from all the participants. Both online and offline surveys were used to collect the data, and the assistant nurses assessed the questionnaires for completeness.

Statistical analyses

Statistical analyses were performed using the Statistical Package for the Social Sciences version 22.0 and Mplus version 8.0. Categorical and continuous variables are expressed as frequencies and proportions and medians with interquartile ranges, respectively. The models were run from one to six classes to determine the optimal number of latent classes. The model fit for LCA included the Akaike information criterion (AIC), Bayesian information criterion (BIC), sample size adjusted BIC (aBIC), entropy, and the Lo-Mendell-Rubin likelihood-ratio test (LMR-LRT). Lower AIC, BIC, and aBIC, higher entropy, and significant LMR-LRT indicate a better model fit.[12,13]

After identifying the optimal latent class model, an analysis of covariance was performed to examine how demographic, clinical characteristics, and functional status differed among the subgroups using the chi-squared and Fisher's exact tests. Subsequently, a multivariate logistic regression model was used to identify the factors associated with different classes, and the results were reported as adjusted odds ratios (ORs) with 95% confidence intervals (CIs). Additionally, a comparison of functional status was performed using one-way analysis of variance (ANOVA). Statistical significance was defined as $P<0.05$.

Patient and public involvement

During the design and preparation of the data collection tool, no study participants have involved in the development of the research question and outcome measures.

RESULTS

Patient characteristics

For peer review only

The demographic and clinical characteristics of the patients are reported in Table 1. A total of 216 patients were available for the analysis, with an average age of 60.3 years (SD=10.4 [range=22-84]). Most of the patients were female (59.7%), were married (92.1%), had received junior high school or below education (70.8%), were retired (38.0%), and had a monthly family income below ¥5000 (76.9%). For cancer-related detail, most of the patients were diagnosed with MM of immunoglobulin G type (55.1%); received induction period of chemotherapy (42.1%); did not take painkillers, antidepressants, or hypnotic drugs (78.2%); and had complications of MM bone disease (44.9%). Additionally, most of them were in the normal range of BMI (63.0%), with an average of 6.8 (SD=1.5), and reported daily step counts lower than 5000 steps (71.3%).

For peer review only

Table 1 Demographic and clinical characteristics of the sample (N=216)

**Variable**	**n (%)**
**Sex**	
Male	87 (40.3)
Female	129 (59.7)
**Marital status**	
Married	199 (92.1)
Separated/divorced/widowed	17 (7.9)
**Educational background**	
Junior high school or below	153 (70.8)
High school	35 (16.2)
University or above	28 (13.0)
**Employment status**	
Employed	14 (6.5)
Medical leave	10 (4.6)
Unemployed	68 (31.5)
Retired	82 (38.0)
Others	42 (19.4)
**Monthly family income (¥)**	
<5000	166 (76.9)
≥5000	50 (23.1)
**MM type**	
IgG type	119 (55.1)
IgA type	79 (36.6)
IgD type	9 (4.2)
Light chain type	7 (3.2)

Others	2 (0.9)
**Disease staging**	
New diagnosis	13 (6.0)
Induction chemotherapy	91 (42.1)
Consolidation chemotherapy	62 (28.7)
Platform stage	27 (12.5)
Unclear	7 (3.2)
Recurrence	16 (7.4)
**Use of painkillers, antidepressants, or hypnotic drugs**	
No	169 (78.2)
Yes	47 (21.8)
**Complication**	
No	5 (2.3)
Multiple myeloma bone disease	97 (44.9)
Impairment of renal function	32 (14.8)
Anemia	71 (32.9)
Hypercalcemia	5 (2.3)
Thrombosis	5 (2.3)
Inflammation	40 (18.5)
Other complications	53 (24.5)
**Treatment**	
Oral drug therapy	8 (3.7)
Chemotherapy	170 (78.7)
Autologous hematopoietic stem cell transplantation	19 (8.8)
Others	19 (8.8)
**BMI**	
<18.5	14 (6.5)
18.5-23.9	136 (63.0)
24-27.9	52 (24.0)
>27.9	14 (6.5)
**Daily step counts**	
<5000	154 (71.3)
≥5000	62 (28.7)

Latent class analysis

The detailed results of the model fitting statistics for one to six classes are presented in Table 2. Compared with other types of model solutions, the three-class solution not only had the best fitting statistical combination (lowest AIC and BIC values), but was also the most parsimonious solution, in addition to the optimal differences in theory and clinic. Therefore, three-class solution was selected as the best solution.

For peer review only

Table 2 Model fit statistics for each of the fitted latent class analysis models

Model	AIC	BIC	aBIC	Entropy	LMR *P* value	BLRT *P* value	Classification probability
2C	1183.08	1220.21	1185.35	0.800	<0.001	<0.001	0.505/0.495
3C	1169.58	1226.96	1173.08	0.710	<0.001	<0.001	0.366/0.342/0.292
4C	1178.47	1256.10	1183.22	0.811	0.491	0.667	0.199/0.287/0.148/0.366
5C	1186.50	1284.38	1192.49	0.786	0.582	1.000	0.292/0.278/0.314/0.019/0.347
6C	1197.35	1315.49	1204.58	0.770	0.332	1.000	0.037/0.032/0.356/0.269/0.292/0.014

AIC, Akaike information criterion; BIC, Bayesian information criterion; aBIC, adjusted Bayesian information criterion; LMR, Lo-Mendell-Rubin; BLRT, bootstrap likelihood ratio test

For peer review only

Subgroup description

The item probabilities of the latent classes are shown in Figure 1. Class 1 was characterized by low item probabilities on all symptom aspects and was labeled as the “low symptom group” (n=79, 36.6%). Class 2 was characterized by elevated item probabilities of anxiety, depression, and pain interference, whereas decreased item probabilities were associated with fatigue and sleep disturbance. Therefore, Class 2 was labelled as the “high anxiety, depression, and pain interference group” (n=74, 34.2%). Patients in Class 3 had low item probabilities on all symptoms and was labeled as the “high symptom group” (n=63, 29.2%).

Demographic and clinical characteristics across the latent classes

Demographic and clinical characteristics of the latent classes are described in Table 3. The results of the chi-squared tests indicated that the proportion of patients assigned to the identified latent classes varied in terms of monthly family income (P=0.001); use of painkillers, antidepressants, or hypnotic drugs (P<0.001); MM bone disease (P<0.001); anemia (P=0.014); other complications (P=0.017); and daily step counts (P=0.043). ANOVA results indicated no significant differences in daily sleep time (P=0.014) among the identified latent classes. No meaningful differential effects were identified for other variables.

Based on the outcomes of univariate analysis, a multinomial logistic regression analysis demonstrated that patients with low monthly family income (OR=3.14, P=0.010) and complications of MM bone disease (OR=2.95, P=0.029) were more likely to belong to Class 2. Additionally, patients using painkillers, antidepressants, or hypnotic drugs (OR=3.68, P=0.012) and with daily step counts less than 5000 steps (OR=2.52, P=0.039) had a higher disposition to Class 3, which was characterized by a high incidence of symptoms. Adequate daily sleep time (OR=0.74, P=0.031) played a protective role in avoiding a high incidence of symptoms (Table 4).

Table 3 Comparisons of demographic and clinical characteristics among the latent classes

Variable	Class 1 (n=79)	Class 2 (n=74)	Class 3 (n=63)	*P* value
**Age (mean ± SD), years**	58.7±10.4	61.5±9.5	60.8±11.4	0.239
**Sex**				0.258
Male	35 (44.3%)	32 (43.2%)	20 (31.7%)	
Female	44 (55.7%)	42 (56.8%)	43 (68.3%)	
**Marital status**				0.320
Married	8 (10.1%)	3 (4.1%)	6 (9.5%)	
Separated/divorced/widowed	71 (89.9%)	71 (95.9%)	57 (90.5%)	
**Educational background**				0.477
Junior high school or below	53 (67.1%)	54 (73.0%)	46 (73.0%)	
High school	12 (15.2%)	14 (18.9%)	9 (14.3%)	
University or above	14 (17.7%)	6 (8.1%)	8 (12.7%)	
**Employment status**				0.814
Employed	6 (7.6%)	5 (6.8%)	3 (4.8%)	
Medical leave	2 (2.5%)	5 (6.8%)	3 (4.8%)	
Unemployed	25 (31.7%)	23 (31.1%)	20 (31.7%)	
Retired	28 (35.4%)	31 (41.8%)	23 (36.5%)	
Others	18 (22.8%)	10 (13.5%)	14 (22.2%)	
**Monthly family income (¥)**				0.001
<5000	50 (63.3%)	64 (86.5%)	52 (82.5%)	
≥5000	29 (36.7%)	10 (13.5%)	11 (17.5%)	
**MM type**				0.797
IgG type	42 (53.1%)	43 (58.1%)	34 (54.0%)	
IgA type	28 (35.4%)	27 (36.4%)	24 (38.1%)	
IgD type	4 (5.1%)	1 (1.4%)	4 (6.3%)	
Light chain type	4 (5.1%)	2 (2.7%)	1 (1.6%)	
Others	1 (1.3%)	1 (1.4%)	0 (0.0%)	
**Disease stage**				0.056

New diagnosis	6 (7.6%)	2 (2.7%)	5 (7.9%)	
Induction chemotherapy	34 (43.1%)	31 (41.9%)	26 (41.3%)	
Consolidation chemotherapy	22 (27.8%)	25 (33.8%)	15 (23.8%)	
Platform stage	14 (17.7%)	7 (9.4%)	6 (9.5%)	
Unclear	2 (2.5%)	4 (5.4%)	1 (1.6%)	
Recurrence	1 (1.3%)	5 (6.8%)	10 (15.9%)	
**Use of painkillers, antidepressants, or hypnotic drugs**				<0.001
No	71 (89.9%)	59 (79.7%)	39 (61.9%)	
Yes	8 (10.1%)	15 (20.3%)	24 (38.1%)	
**Complication**				
No	2 (2.5%)	1 (1.4%)	2 (3.2%)	0.860
Multiple myeloma bone disease	20 (25.3%)	41 (55.4%)	36 (57.1%)	<0.001
Impairment of renal function	9 (11.4%)	9 (12.2%)	14 (22.2%)	0.143
Anemia	17 (21.5%)	26 (35.1%)	28 (44.4%)	0.014
Hypercalcemia	2 (2.5%)	0 (0.0%)	3 (4.8%)	0.197
Thrombosis	1 (1.3%)	3 (4.1%)	1 (1.6%)	0.531
Inflammation	16 (20.3%)	16 (21.6%)	8 (12.7%)	0.355
Other complications	28 (35.4%)	13 (17.6%)	12 (19.0%)	0.017
**Treatments**				0.804
Oral drug therapy	3 (3.8%)	4 (5.4%)	1 (1.6%)	
Chemotherapy	64 (81.0%)	55 (74.3%)	51 (81.0%)	
Autologous hematopoietic stem cell transplantation	5 (6.3%)	9 (12.2%)	5 (7.9%)	
Others	7 (8.9%)	6 (8.1%)	6 (9.5%)	
**BMI**				0.198
<18.5	6 (7.6%)	2 (2.7%)	6 (9.5%)	
18.5-23.9	50 (63.3%)	52 (70.3%)	34 (54.0%)	
24-27.9	20 (25.3%)	13 (17.6%)	19 (30.2%)	
>27.9	3 (3.8%)	7 (9.5%)	4 (6.3%)	
**Daily sleep time (mean ± SD), hours**	7.2±1.4	6.7±1.4	6.5±1.5	0.014
**Daily step counts**				0.043
<5000	49 (62.0%)	54 (73.0%)	51 (81.0%)	
≥5000	30 (38.0%)	20 (27.0%)	12 (19.0%)	

Table 4 Potential predictors of latent class membership

**Characteristics**	**Class 2 (n=74)**		***P* value**	**Class 3 (n=63)**		***P* value**
	**OR**	**95% C.I.**		**OR**	**95% C.I.**	
**Monthly family income**						
<5000	3.14	1.32-7.44	0.010	2.33	0.95-5.70	0.064
≥5000	Ref			Ref		
**Use of painkillers,**						

For peer review only

**antidepressants or hypnotic drugs**						
No	Ref			Ref		
Yes	1.66	0.59-4.68	0.338	3.68	1.34-10.11	0.012
**Complication (osteopathy)**						
No	Ref			Ref		
Yes	2.95	1.11-7.83	0.029	2.81	0.98-8.07	0.055
**Complication (multiple myeloma bone disease)**						
No	Ref			Ref		
Yes	1.52	0.58-4.00	0.398	2.10	0.75-5.86	0.158
**Other complications**						
No	Ref			Ref		
Yes	0.75	0.28-2.00	0.563	1.04	10.36-3.00	0.946
**Daily sleep time**	0.83	0.64-1.07	0.143	0.74	0.57-0.97	0.031
**Daily step counts**						
≥5000	Ref			Ref		
<5000	1.59	0.74-3.42	0.234	2.52	1.05-6.05	0.039

OR, odds ratio; 95% CI, 95% confidence interval of OR

For peer review only

Between-group differences in functional status across the latent classes

A comparison of functional status across the latent classes is reported in Table 5. The T-scores among physical function, ability to participate in social roles and activities, and cognitive function were relatively low in the sample according to the score manual of PROMIS-57 (44.4±8.1, 49.4±8.6, and 45.2±8.2, respectively), in which physical function was slightly impaired when compared with the general population. Significant differences were identified among the three classes, in which Class 1 scored the highest in all functional dimensions, whereas Class 3 scored the lowest ($P<0.05$).

Table 5 Functional status across the identified latent classes (mean ± SD)

Domain	Class 1 (n=79)	Class 2 (n=74)	Class 3 (n=63)	*P* value
Physical function	50.2±6.7	44.0±4.1	37.8±7.9	<0.001
Ability to participate in social roles and	56.0±8.0	49.0±4.1	41.6±6.0	<0.001

activities				
Cognitive function	50.5±8.6	44.8±5.5	39.0±5.2	<0.001

Discussion

To the best of our knowledge, this is the first study to describe the homogeneous symptoms and associated factors in patients with MM using routinely captured PROs. Our findings highlighted those patients with MM differed substantially in average stability. A high prevalence of burdensome symptoms was found in this population during treatment, and 63.4% of them reported varied symptom burdens, which deserves clinical attention.

The current study identified the target population with the most severe symptoms. Classes 2 and 3 represented groups with greater symptom burden, in which Class 3 patients were at a higher risk. Depression, fatigue, and pain were identified as the most common symptoms in this study, which is consistent with the broader literature.[14,15] Boland et al.[14] reported that fatigue and pain in the bone were the most common symptoms in patients receiving treatment for MM. A study focused on patients with stable but advanced-stage MM also confirmed that fatigue and pain were the most frequent symptoms.[15] Furthermore, Naegele et al.[16] reported that fatigue, diarrhea, and decreased appetite were the most frequent and distressing physical symptoms in patients with MM treated with autologous stem cell transplantation, and these symptoms differed substantially before and after treatment. This discrepancy might be caused by differences in the treatment of patients received. According to a systematic review summarizing evidence of symptoms in patients with MM, pain, fatigue, sleep issues, and depression were the most frequently examined symptoms in related studies.[17] The four most prevalent symptoms in this population were fatigue, constipation, pain, and tingling sensation, ranging from 53.4% to almost all of the included studies.[18] Anxiety and depression have been reported to be the most common psychological symptoms. Therefore, demographics and treatment need to be considered in patients with MM.

For peer review only

The demographic and cancer-related factors associated with different symptom profiles were identified in this study. An increase in age did not demonstrate a significant between-group difference. The median age in this sample was 60.3 years, which was relatively younger than the average age of MM (M=73). Therefore, future studies should validate these results in older patients. Complications of MM bone disease were predictive factors for high anxiety, depression, and pain interference. These findings are consistent with those of a previous study conducted by Ebraheem et al.[19], which suggested that increased comorbidities and myeloma-related bone and kidney damage were associated with a higher rate of moderate to severe symptoms. Furthermore, depressive symptoms increase the incidence of a higher number of comorbidities in patients with MM aged ≥65 years.[20]

Similar to the result of a prior study, poor socioeconomic status was associated with increased symptoms of pain in our study, suggesting that these patients may require additional support.[19] Low monthly family income was correlated with severe symptoms, especially in the anxiety, depression, and pain domains. Lower income and unemployment have been reported as risk factors for financial burden as a direct result of cancer and related treatment and can be referred to as financial toxicity.[21] Previous evidence has demonstrated that patients with MM have substantial areas of concern about financial burdens (44%) and inability to perform daily activities (33%) and felt unhappy (41%). In addition, 36% reported pain and 15% reported depression,[22] addressing the need for multidisciplinary psychosocial support. According to Ramsenthaler et al., many patients with MM were burdened by financial worries, almost 50% of patients with MM reported worry about dying, and almost 80% had financial difficulties, of which 43.3% were severe.[17] Psychosocial stressors, including depression, are considered to cause high resistance to cell death, compromised immunity, and inflammatory and oxidative stress, which may affect cancer progression.[23] Additionally, these symptoms are associated with longer hospitalizations, low medication adherence, and suicide.[23] Therefore, patients with poor socioeconomic status deserve more attention, especially those with psychosocial symptoms. The

For peer review only

use of painkillers, antidepressants, or hypnotic drugs and low physical activity were significant predictors of a high symptom burden in patients with MM. Patients with these medicinal uses are more likely to experience severe pain, depression, and sleep issues**.** Therefore, these patients require attention.

Consistent with the results of previous studies, patients with burdensome symptoms were more likely to report functional limitation than patients without burdensome symptoms.[15,17,24] For example, symptoms such as fatigue and pain have a negative effect on physical function and can even cause treatment discontinuation.[15,24] In our study, patients' physical function was slightly impaired when compared with the general population, whereas no significant decrease was identified in social and cognitive aspects. The results were not consistent with those reported by Ramsenthaler et al.[17,] suggesting that severely decreased social, physical, and cognitive functions were common in patients with MM. Differences in age might have contributed to these disparities, since relatively younger patients were included in this study. According to a national report in China, the mean age of patients diagnosed with MM was 58 years, which was approximately 10 years younger than that of Caucasians.[25] Since relatively young patients were included in this study, future studies should validate whether the findings can be generalized to other older patients with MM.

Limitations of the study

This study has some limitations. First, this was a cross-sectional study that failed to explore the symptoms of trajectories over time. Second, we only included two single centers, and the results need to be validated in multicenter studies. Moreover, the severity of the symptoms may have been underestimated, as some patients who were significantly ill to participate may not have participated in the survey. Additional studies are required to incorporate more diverse sampling to fill ongoing knowledge gaps in this field.

For peer review only

Future Studies

In future studies, a longitudinal study design should be considered first to present the dynamic changes in patients' symptom characteristics over time and course of disease. Second, multiple data collection methods (such as paper, Internet, or telephone collection) should be innovated to protect the integrity of the data. In addition, a multicenter clinical study is proposed to expand the use of the PROMIS-57 measurement tool in real-world patient care. Therefore, it is necessary to implement symptom management based on PROs in heterogeneous populations.

Conclusion

Three distinct symptom profiles were observed in patients with MM during treatment. Patients with low monthly family income and complications of MM bone disease were more likely to report high levels of anxiety, depression, and pain interference. Taking painkillers, antidepressants, or hypnotic drugs, with daily step counts less than 5000, and with functional limitation increase patients' risk for high symptoms. The identification of patients with high symptom management needs should focus on assessing demographic and clinical characteristics and functional status so that interventions can be designed for high-risk individuals.

Acknowledgements

We thank the study sites, which enabled us to recruit the patients required for this study.

Contributors

CY1, CY2, and TC designed the study. CY1, NZ, GP, and WY are the principal investigators; TC and CY1 wrote the manuscript; CY1, TC, and TZ are in charge of statistical analysis and all authors reviewed and contributed to the manuscript. All authors critically revised the manuscript and gave final approval.

For peer review only

Funding

This work was supported by the Science and Technology Project of Guilin City (20210227-8-5) and Project of the Guangxi Education Department (2021KY0521).

Competing interests

The authors declare no competing interests.

Patient consent for publication

Not required.

Ethics approval

Ethical approval was obtained from the Institutional Review Board of the Second Affiliated Hospital of Guilin Medical University and all study sites (No. GLKJJH010).

Data availability statement

Data are available upon reasonable request. The data that support the findings of this study are available on request from the corresponding author.

ORCID ID

Changrong Yuan, http://orcid.org/ 0000-001-8480-2569

For peer review only

REFERENCES

1 Rosenberg PS, Barker KA, Anderson WF. Future distribution of multiple myeloma in the United States by sex, age, and race/ethnicity. *Blood*, 2015;125(2):410-412.

2 Kiely F, Cran A, Finnerty D, et al. Self-Reported Quality of Life and Symptom Burden in Ambulatory Patients With Multiple Myeloma on Disease-Modifying. *Am J Hosp Palliat Me*, 2017;34:671-676.

3 Ramsenthaler C, Osborne TR, Gao W, et al. The impact of disease-related symptoms and palliative care concerns on health-related quality of life in multiple myeloma: a multi-centre study. *BMC Cancer*, 2016;16:427.

4 Kamal M, Wang XS, Shi Q, et al. Symptom burden and its functional impact in patients with "symptomatic" relapsed or refractory multiple myeloma. *Support Care Cancer*, 2021;29:467-475.

5 El-Jawahri A, Chen YB, Brazauskas R, et al. Impact of pre-transplant depression on outcomes of allogeneic and autologous hematopoietic stem cell transplantation. *Cancer*, 2017;123(10):1828-1838.

6 Cohen MZ, Rozmus CL, Mendoza TR, et al. Symptoms and quality of life in diverse patients undergoing hematopoietic stem cell transplantation. *J Pain Symptom Manag*, 2012;44(2):168-180.

7 Sparano F, Cavo M, Niscola P, et al. Patient-reported outcomes in relapsed/refractory multiple myeloma: a systematic review. *Support Care Cancer*, 2018;26(7):2075-2090.

8 Rahman AF, Manatunga A, Guo Y, et al. A latent class analysis of PTSD symptoms among inner city primary care patients. *J Psychiatr Res*, 2018;98:1-8.

9 Rimehaug SA, Kaat AJ, Nordvik JE, et al. Psychometric properties of the PROMIS-57

For peer review only

questionnaire, Norwegian version. *Qual Life Res*, 2022;31:269-280.

10 Tang E, Ekundayo O, Peipert JD, et al. Validation of the patient-reported outcomes measurement Information system (PROMIS)-57 and -29 item short forms among kidney transplant recipients. *Qual Life Res*, 2019;28(3):815-827.

11 Iverson GL, Marsh JM, Connors EJ, et al. Normative Reference Values, Reliability, and Item-Level Symptom Endorsement for the PROMIS® v2.0 Cognitive Function-Short Forms 4a, 6a and 8a. *Arch Clin Neuropsych*, 2021;7:1341-1349.

12 Nylund KL, Asparouhov T, Muth´en BO. Deciding on the number of classes in latent class analysis and growth mixture modeling: a Monte Carlo simulation study. *Struct Equ Model Multidiscip J*, 2007;14(4):535-569.

13 Watanabe M, Yamauchi K. Subtypes of overtime work and nurses' fatigue, mental status, and work engagement: A latent class analysis of Japanese hospital nurses. *J Adv Nurs*, 2019;75(10):2122-2132.

14 Boland E, Eiser C, Ezaydi Y, et al. Living with advanced but stable multiple myeloma: a study of the symptom burden and cumulative effects of disease and intensive (hematopoietic stem cell transplant-based) treatment on health-related quality of life. *J Pain Symptom Manage*, 2013;46(5):671-680.

15 Jordan K, Proskorovsky I, Lewis P, et al. Effect of general symptom level, specific adverse events, treatment patterns, and patient characteristics on health-related quality of life in patients with multiple myeloma: results of a European, multicenter cohort study. *Support Care Cancer*, 2014;22(2):417-426.

16 Naegele M, Kirsch M, Ihorst G, et al. Symptom experience of multiple myeloma (syMMex) patients treated with autologous stem cell transplantation following high-dose melphalan: a

For peer review only

descriptive longitudinal study. *Support Care Cancer*, 2017;26(3):833-841.

17 Ramsenthaler C, Kane P, Gao W, et al. Prevalence of symptoms in patients with multiple myeloma: a systematic review and meta-analysis. *Eur J Haematol*, 2016;97(5):416-429.

18 Weisel K, Dimopoulos M, Song KW, et al. Pomalidomide and low-dose dexamethasone improves health-related quality of life and prolongs time to worsening in relapsed/refractory patients with multiple myeloma enrolled in the MM-003 randomized phase III trial. *Clin Lymphoma Myeloma Leuk*, 2015;15:519-530.

19 Ebraheem MS, Seow H, Balitsky AK, et al. Trajectory of Symptoms in Patients Undergoing Autologous Stem Cell Transplant for Multiple Myeloma: A Population-Based Cohort Study of Patient-Reported Outcomes. *Clin Lymphoma Myeloma Leuk*, 2021;21(9):e714-e721. https://doi.org/10.1016/j.clml.2021.05.002

20 Alobaidi A, Nabulsi NA, Talon B, et al. Depressive symptoms, mental health-related quality of life, and survival among older patients with multiple myeloma. *Support Care Cancer*. 2020;28:4097-4106.

21 Smith GL, Lopez-Olivo MA, Advani PG, et al. Financial burdens of cancer treatment: A systematic review of risk factors and outcomes. *J National Compr Canc Ne*, 2019;17(10):1184-1192.

22 Richter J, Sanchez L, Biran N, et al. Prevalence and Survival Impact of Self-Reported Symptom and Psychological Distress Among Patients With Multiple Myeloma. *Clin Lymphoma Myeloma Leuk*, 2021;21(3):e284-e289.

23 Bortolato B, Hyphantis TN, Valpione S, et al. Depression in cancer: the many biobehavioral pathways driving tumor progression. *Cancer Treat Rev*, 52:58-70.

24 Wang XS, Shi Q, Mendoza TR, et al. Minocycline for symptom reduction in patients with

For peer review only

multiple myeloma during maintenance therapy: a phase II placebo-controlled randomized trial. *Support Care Cancer*, 2021;29(10):6099-6107.

25 Wang S, Xu L, Feng J, et al. Prevalence and Incidence of Multiple Myeloma in Urban Area in China: A National Population-Based Analysis. *Front Oncol*, 2020;9:1513.

For peer review only

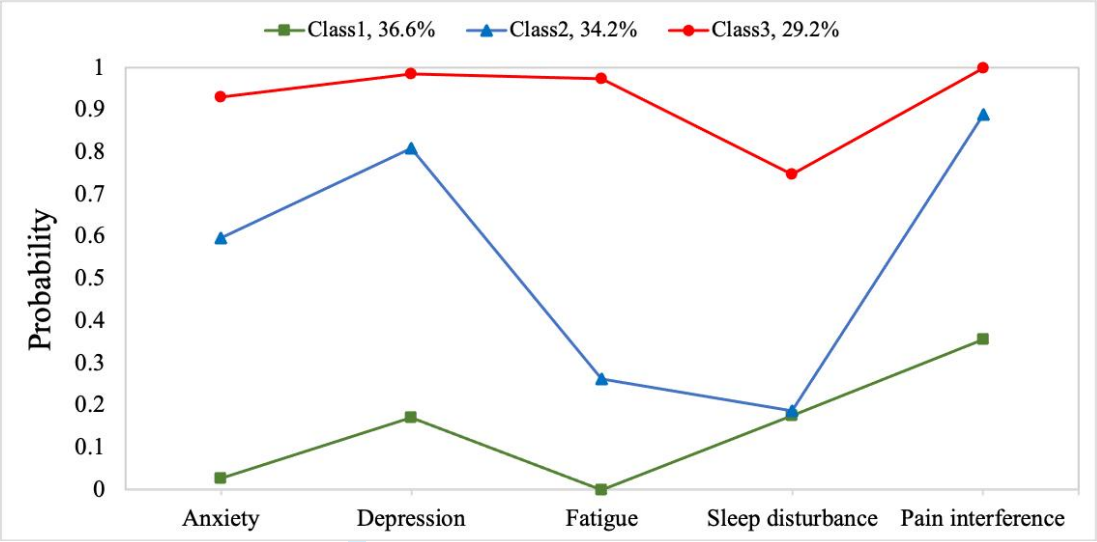

Figure 1 The three-class latent profile analysis model

For peer review only

STROBE Statement—Checklist of items that should be included in reports of ***cross-sectional studies***

	Item No	Recommendation	Page No
**Title and abstract**	1	(*a*) Indicate the study's design with a commonly used term in the title or the abstract	0-1
		(*b*) Provide in the abstract an informative and balanced summary of what was done and what was found	1-2
**Introduction**			
Background/rationale	2	Explain the scientific background and rationale for the investigation being reported	3-4
Objectives	3	State specific objectives, including any prespecified hypotheses	4
**Methods**			
Study design	4	Present key elements of study design early in the paper	4
Setting	5	Describe the setting, locations, and relevant dates, including periods of recruitment, exposure, follow-up, and data collection	4
Participants	6	(*a*) Give the eligibility criteria, and the sources and methods of selection of participants	4
Variables	7	Clearly define all outcomes, exposures, predictors, potential confounders, and effect modifiers. Give diagnostic criteria, if applicable	4-6
Data sources/ measurement	8*	For each variable of interest, give sources of data and details of methods of assessment (measurement). Describe comparability of assessment methods if there is more than one group	4-6
Bias	9	Describe any efforts to address potential sources of bias	-
Study size	10	Explain how the study size was arrived at	4
Quantitative variables	11	Explain how quantitative variables were handled in the analyses. If applicable, describe which groupings were chosen and why	5-6
Statistical methods	12	(*a*) Describe all statistical methods, including those used to control for confounding	6
		(*b*) Describe any methods used to examine subgroups and interactions	6
		(*c*) Explain how missing data were addressed	6
		(*d*) If applicable, describe analytical methods taking account of sampling strategy	6
		(*e*) Describe any sensitivity analyses	-
**Results**			
Participants	13*	(a) Report numbers of individuals at each stage of study—eg numbers potentially eligible, examined for eligibility, confirmed eligible, included in the study, completing follow-up, and analysed	6-7
		(b) Give reasons for non-participation at each stage	-
		(c) Consider use of a flow diagram	-
Descriptive data	14*	(a) Give characteristics of study participants (eg demographic, clinical, social) and information on exposures and potential confounders	7-12
		(b) Indicate number of participants with missing data for each variable of interest	-
Outcome data	15*	Report numbers of outcome events or summary measures	7-12
Main results	16	(*a*) Give unadjusted estimates and, if applicable, confounder-adjusted estimates and their precision (eg, 95% confidence interval). Make clear which confounders were adjusted for and why they were included	-

For peer review only

For peer review only - http://bmjopen.bmj.com/site/about/guidelines.xhtml

		(*b*) Report category boundaries when continuous variables were categorized	8-9
		(*c*) If relevant, consider translating estimates of relative risk into absolute risk for a meaningful time period	-
Other analyses	17	Report other analyses done—eg analyses of subgroups and interactions, and sensitivity analyses	9-12
**Discussion**			
Key results	18	Summarise key results with reference to study objectives	13-16
Limitations	19	Discuss limitations of the study, taking into account sources of potential bias or imprecision. Discuss both direction and magnitude of any potential bias	15
Interpretation	20	Give a cautious overall interpretation of results considering objectives, limitations, multiplicity of analyses, results from similar studies, and other relevant evidence	13-16
Generalisability	21	Discuss the generalisability (external validity) of the study results	16
**Other information**			
Funding	22	Give the source of funding and the role of the funders for the present study and, if applicable, for the original study on which the present article is based	20

*Give information separately for exposed and unexposed groups.

Note: An Explanation and Elaboration article discusses each checklist item and gives methodological background and published examples of transparent reporting. The STROBE checklist is best used in conjunction with this article (freely available on the Web sites of PLoS Medicine at http://www.plosmedicine.org/, Annals of Internal Medicine at http://www.annals.org/, and Epidemiology at http://www.epidem.com/). Information on the STROBE Initiative is available at www.strobe-statement.org.

For peer review only

For peer review only - http://bmjopen.bmj.com/site/about/guidelines.xhtml

Classification of symptom subtypes in patients with multiple myeloma during treatment: A cross-sectional survey study in China

Journal:	*BMJ Open*
Manuscript ID	bmjopen-2022-066467.R1
Article Type:	Original research
Date Submitted by the Author:	26-Nov-2022
Complete List of Authors:	Yu, Chunfang; The Second Affiliated Hospital of Guilin Medical University<br>Cai, Tingting; Fudan University School of Nursing<br>Zhou, Tingting; Fudan University School of Nursing<br>Zeng, Ning; Affiliated Hospital of Guilin Medical University<br>Liang, Xin; Guangxi Normal University<br>Pan, Guihua; Affiliated Hospital of Guilin Medical University<br>Ouyang, Wei; Peking Union Medical College Hospital, Department of Nursing<br>Yuan, Changrong; Fudan University School of Nursing,
**Primary Subject Heading**:	Nursing
Secondary Subject Heading:	Oncology, Research methods, Haematology (incl blood transfusion), Mental health
Keywords:	CHEMOTHERAPY, Lymphoma < HAEMATOLOGY, Myeloma < HAEMATOLOGY, Adult oncology < ONCOLOGY

I, the Submitting Author has the right to grant and does grant on behalf of all authors of the Work (as defined in the below author licence), an exclusive licence and/or a non-exclusive licence for contributions from authors who are: i) UK Crown employees; ii) where BMJ has agreed a CC-BY licence shall apply, and/or iii) in accordance with the terms applicable for US Federal Government officers or employees acting as part of their official duties; on a worldwide, perpetual, irrevocable, royalty-free basis to BMJ Publishing Group Ltd ("BMJ") its licensees and where the relevant Journal is co-owned by BMJ to the co-owners of the Journal, to publish the Work in this journal and any other BMJ products and to exploit all rights, as set out in our licence.

The Submitting Author accepts and understands that any supply made under these terms is made by BMJ to the Submitting Author unless you are acting as an employee on behalf of your employer or a postgraduate student of an affiliated institution which is paying any applicable article publishing charge ("APC") for Open Access articles. Where the Submitting Author wishes to make the Work available on an Open Access basis (and intends to pay the relevant APC), the terms of reuse of such Open Access shall be governed by a Creative Commons licence – details of these licences and which Creative Commons licence will apply to this Work are set out in our licence referred to above.

Other than as permitted in any relevant BMJ Author's Self Archiving Policies, I confirm this Work has not been accepted for publication elsewhere, is not being considered for publication elsewhere and does not duplicate material already published. I confirm all authors consent to publication of this Work and authorise the granting of this licence.

For peer review only

Title Page

Manuscript category: research paper

Title: Classification of symptom subtypes in patients with multiple myeloma during treatment: A cross-sectional survey study in China

Authors and Affiliation:

Authors: Chunfang Yu[1#], MD; Tingting Cai[2#], PhD; Tingting Zhou[2], MD; Ning Zeng[3], MD; Xin Liang[4], PhD; Guihua Pan[3], RN; Wei Ouyang[5], RN; Changrong Yuan[2*], PhD

Note: Chunfang Yu and Tingting Cai made equal contributions to this manuscript and should be regarded as joint first author.

Affiliations:

[1]Department of Hematopathology, the Second Affiliated Hospital of Guilin Medical University, Guilin, China

[2]School of Nursing, Fudan University, Shanghai, China

[3]Affiliated Hospital of Guilin Medical University, Guilin, China

[4]School of Mathematics and Statistics, Guangxi Normal University, Guilin, China

[5]Department of Nursing, Peking Union Medical College Hospital, Beijing, China

Correspondence: Changrong Yuan, PhD, RN, FAAN, Fudan University, School of Nursing, 305 Fenglin Road Shanghai 200032, China (yuancr@fudan.edu.cn), Tel: 02164431803

Word counts: 3,547 words

For peer review only

Abstract

Objectives: To classify subgroups of cancer-related symptoms in patients with multiple myeloma (MM) during treatment and examine between-group differences in demographic and clinical characteristics in addition to functional status.

Design: Cross-sectional survey study

Setting: Hematology department of two tertiary hospitals affiliated with Guilin Medical University in China

Participants: Using a convenience sampling method, questionnaires were distributed to patients with MM visiting two hospitals in Guilin, China.

Interventions: The patients were categorized into subgroups based on cancer-related symptoms using a latent class analysis. An analysis of covariance was performed to examine how demographic and clinical characteristics and functional status differed among the subgroups.

Results: In total, 216 patients completed the survey, with an average age of 60.3 years. A three-class solution was identified: low symptom burden group (Class 1, 36.6%), moderate symptom burden group (Class 2, 34.2%), and high symptom burden group (Class 3, 29.2%). Patients with low monthly family income (odds ratio [OR]=3.14, *P*=0.010) and complications of MM bone disease (OR=2.95, *P*=0.029) were more likely to belong to Class 2. The predictors of high burden symptoms were treatment with painkillers, antidepressants, or hypnotic drugs (OR=3.68, *P*=0.012) and <5000 daily step counts (OR=2.52, *P*=0.039) in Class 3. Functional status was correlated with symptom burden, with patients in Classes 3 and 1 reporting significantly higher and lower functional status, respectively (*P*<0.05).

Conclusions: Patients with MM experienced varying degrees of symptoms during treatment. The

For peer review only

identification of patients with high symptom burden management should focus on the assessment of demographic and clinical characteristics, in addition to functional status.

Keywords: multiple myeloma, patient-reported outcomes, symptoms, function, latent class analysis

Strengths and limitations of this study

- A latent class analysis was used to categorize cancer-related symptoms in Chinese patients undergoing treatment for multiple myeloma.
- This study assessed between-group differences in the demographic and clinical characteristics and functional status of patients with multiple myeloma.
- The Patient-Reported Outcomes Information System (PROMIS)-57 and the PROMIS Cognitive Function Short Form were used as research tools.
- This study involved a limited number of treatment centers.
- Patient clinical characteristics did not include the evaluation of treatment response.

For peer review only

INTRODUCTION

Multiple myeloma (MM) is the second most common hematological cancer worldwide [1]. Although still incurable, advancements in the management of MM have extended survival, and MM is now considered a chronic disease. MM is primarily a cancer of old age with a median age at diagnosis of 73 years [1]. Older patients have a higher rate of comorbid chronic and progressive diseases than younger patients, and, compared to younger patients, a more careful approach is required to older patients during MM treatment to manage multiple treatment-related issues, including symptoms and functional limitations [2]. Patients with MM frequently report symptoms, including pain, fatigue, and depression, after diagnosis and throughout treatment [3]. With increasing survival rates, increased attention has focused on symptom control in this population.

Although advancements in MM treatment have been extensively reported, little is known about the symptoms of patients with MM during treatment [4-6]. Monitoring symptoms complements traditional laboratory testing and contributes to the identification of individuals who require supportive interventions. The effects of MM and its treatment may have an effect on patients' physical and emotional well-being, in addition to their social function. There is a paucity of data regarding the symptoms experienced by patients with MM, particularly those undergoing therapy. Symptoms may affect cancer treatment options and outcomes. Understanding individual symptom experiences is important to identify patients who are at risk of manifesting severe symptoms.

Evidence suggests that direct patient reports are the best way to capture information on patient experience of disease and related treatment [2, 6, 7]. Patient-reported outcomes (PROs) are strongly recommended for the measurement of symptoms and treatment effectiveness in patients with MM [7]. Specifically, among patients undergoing therapy, PROs can provide a better understanding of the magnitude and temporal trends of symptoms from the perspective of individual patients. Additionally, using PROs to capture patient symptoms is necessary to perform value-based and patient-centered care and to enable more accurate symptom evaluation [7].

To date, the heterogeneity of symptoms has not been captured among patients with MM.

For peer review only

Patients experience multiple symptoms that potentially interact with each other and that occur in clusters of clinically distinct subgroups. Statistical advancements in modeling contribute to the identification of subtypes and may be useful in distinguishing patients at risk. Latent class analysis (LCA) is a person-oriented approach used to classify individuals based on their symptom profiles and estimate distinct patterns [8].

There is no existing study to explore symptom profiles resulting from MM and its treatment in this population. Therefore, this study aimed to classify subgroups of patients with MM by incorporating PRO instruments and to examine between-group differences in demographic and clinical characteristics, in addition to functional status.

METHODS

Study design and sample

This study adopted a cross-sectional study design and adhered to the Strengthening The Reporting of OBservational Studies in Epidemiology guidelines. Convenience sampling was used to recruit patients with MM visiting the hematology department of two tertiary hospitals affiliated with Guilin Medical University in China between July and December 2021. Eligible patients were aged >18 years, diagnosed with primary MM, hospitalized for treatment, and able to speak and write Mandarin Chinese. Patients with other types of cancer or mental illnesses were excluded from this study.

Study instruments

Demographic and clinical data

Demographic and socioeconomic data included age, sex, marital status, educational background, employment status, monthly family income, medical insurance, daily sleep duration, and daily step counts. The MM type, disease stage, drug usage, complications, treatments, and body mass index (BMI) were retrieved from the medical records.

For peer review only

Patient-Reported Outcomes Measurement Information System (PROMIS)-57

Symptom experiences were measured using the Patient-Reported Outcomes Measurement Information System (PROMIS)-57 instrument. The PROMIS-57 consists of 57 items and includes five symptoms (anxiety, depression, fatigue, sleep disturbance, pain interference) and two functional domains (physical function, ability to participate in social roles and activities), along with one additional pain intensity item [9]. The instrument adopts a Likert scale (0–5) to assess the severity of symptoms or functional limitation, except for pain intensity, which has 11 response options (ranging from 0 to 10). The raw score of all outcome scores ranged from 4 to 20 and was converted into T-scores with a mean of 50 and a standard deviation (SD) of 10 in the scoring manual [10]. A higher score indicates poor functional status; however, for the symptom domain, this is reversed, with higher scores reflecting a high symptom burden [9]. Cronbach's α coefficient ranged from 0.85 to 0.95 in this study.

PROMIS-Cognitive Function Short Form

The four-item PROMIS-Cognitive Function Short Form measures assessed cognitive function over the preceding 7 days. Each question was given a response of 1 ("never") to 5 ("very often") [11]. The total raw scores ranged from 4 to 20 and were converted into T-scores. Similar to the PROMIS-57, lower T-scores indicate greater subjective cognitive difficulty. The Cronbach α for the scale was 0.93.

Data collection

The study protocol was approved by the Ethics Committee of the Second Affiliated Hospital of Guilin Medical University (No. GLKJJH010). Eligible patients were approached and provided with information about the study by assistant nurses. Written informed consent was obtained from all the participants. Both online and offline surveys were used to collect the data, and the assistant nurses assessed the questionnaires for completeness.

For peer review only

Statistical analyses

Statistical analyses were performed using the Statistical Package for the Social Sciences version 22.0 and Mplus version 8.0. Categorical and continuous variables are expressed as frequencies and proportions and medians with interquartile ranges, respectively. The models were run from one to six classes to determine the optimal number of latent classes. The model fit for LCA included the Akaike information criterion (AIC), Bayesian information criterion (BIC), sample size adjusted BIC (aBIC), entropy, and the Lo-Mendell-Rubin likelihood-ratio test (LMR-LRT). Lower AIC, BIC, and aBIC, higher entropy, and significant LMR-LRT indicate a better model fit [12,13].

After identifying the optimal latent class model, the symptom probabilities of identified subgroups were depicted by a line chart to present the characteristics of each group. To further identify between-group difference among the subgroups, an analysis of covariance was performed to examine how demographic, clinical characteristics, and functional status differed among the subgroups using the chi-squared and Fisher's exact tests. Subsequently, a multivariate logistic regression model was used to identify the factors associated with different classes, and the results were reported as adjusted odds ratios (ORs) with 95% confidence intervals (CIs). Additionally, a comparison of functional status was performed using one-way analysis of variance (ANOVA). Statistical significance was defined as $P<0.05$.

Patient and public involvement

During the design and preparation of the data collection tool, no study participants were involved in the development of the research question or the outcome measures.

RESULTS

Patient characteristics

The demographic and clinical characteristics of the patients are reported in Table 1. In total, 216 patients were available for the analysis, with an average age of 60.3 (SD=10.4 [range=22–84])

For peer review only

years. Most of the patients were female (59.7%), married (92.1%), and retired (38.0%), with an educational level of junior high school or below (70.8%) and a monthly family income below ¥5000 (76.9%). In terms of clinical characteristics, most patients had IgG MM (55.1%), were undergoing chemotherapy (70.8%), and had multiple concurrent complications of treatment (97.7%). Regarding treatment options, most patients underwent chemotherapy (78.7%) and did not require analgesia, antidepressants, or hypnotics before the diagnosis (97.7%). Most patients had a BMI within the normal range (63.0%) and reported daily step counts <5000 steps (71.3%).

Table 1 Demographic and clinical characteristics of the sample (n=216)

Variable	n (%)
**Sex**	
Male	87 (40.3)
Female	129 (59.7)
**Marital status**	
Married	199 (92.1)
Separated/divorced/widowed	17 (7.9)
**Educational background**	
Junior high school or below	153 (70.8)
High school	35 (16.2)
University or above	28 (13.0)
**Employment status**	
Employed	14 (6.5)
Medical leave	10 (4.6)
Unemployed	68 (31.5)
Retired	82 (38.0)
Others	42 (19.4)
**Monthly family income (¥)**	
<5000	166 (76.9)
≥5000	50 (23.1)
**MM type**	
IgG type	119 (55.1)
IgA type	79 (36.6)
IgD type	9 (4.2)
Light chain type	7 (3.2)
Others	2 (0.9)
**Disease staging**	
New diagnosis	13 (6.0)
Induction chemotherapy	91 (42.1)
Consolidation chemotherapy	62 (28.7)

For peer review only

Platform stage	27 (12.5)
Unclear	7 (3.2)
Recurrence	16 (7.4)
**Use of painkillers, antidepressants, or hypnotic drugs**	
No	169 (78.2)
Yes	47 (21.8)
**Number of complications**	
0	5 (2.3)
1	145 (67.1%)
2	44 (20.4%)
≥3	22 (10.2%)
**Types of complications**	
Multiple myeloma bone disease	97 (44.9)
Impairment of renal function	32 (14.8)
Anemia	71 (32.9)
Hypercalcemia	5 (2.3)
Thrombosis	5 (2.3)
Inflammation	40 (18.5)
Other complications	53 (24.5)
**Treatment**	
Oral drug therapy	8 (3.7)
Chemotherapy	170 (78.7)
Autologous hematopoietic stem cell transplantation	19 (8.8)
Others	19 (8.8)
**BMI**	
<18.5	14 (6.5)
18.5–23.9	136 (63.0)
24–27.9	52 (24.0)
>27.9	14 (6.5)
**Daily step counts**	
<5000	154 (71.3)
≥5000	62 (28.7)

Latent class analysis

The detailed results of the model fitting statistics for one to six classes are presented in Table 2. Among the types of model solutions, the three-class solution had the best fit (lowest AIC and BIC values), high entropy, significant *P* values for LMR and BLRT, and optimal clinical interpretability of each latent class. Therefore, the three-class solution was selected as the optimal solution.

For peer review only

Table 2 Model fit statistics for each of the fitted latent class analysis models

Model	AIC	BIC	aBIC	Entropy	LMR *P* value	BLRT *P* value	Classification probability
2C	1183.08	1220.21	1185.35	0.800	<0.001	<0.001	0.505/0.495
3C	1169.58	1226.96	1173.08	0.710	<0.001	<0.001	0.366/0.342/0.292
4C	1178.47	1256.10	1183.22	0.811	0.491	0.667	0.199/0.287/0.148/0.366
5C	1186.50	1284.38	1192.49	0.786	0.582	1.000	0.292/0.278/0.314/0.019/0.347
6C	1197.35	1315.49	1204.58	0.770	0.332	1.000	0.037/0.032/0.356/0.269/0.292/0.014

AIC, Akaike information criterion; BIC, Bayesian information criterion; aBIC, adjusted Bayesian information criterion; LMR, Lo-Mendell-Rubin; BLRT, bootstrap likelihood ratio test

For peer review only

Classification of symptoms and functional status

The item probabilities of the latent classes are shown in Supplementary Figure 1. Class 1 was characterized by low item probabilities for all symptom aspects and was labeled as the “low symptom burden group” (n=79, 36.6%). Class 2 was characterized by elevated item probabilities for anxiety, depression, and pain interference, whereas decreased item probabilities were associated with fatigue and sleep disturbance. Therefore, Class 2 was labeled as the “moderate symptom burden group” (n=74, 34.2%). Class 3 was characterized by high item probabilities for all symptoms and was labeled as the “high symptom burden group” (n=63, 29.2%).

Between-group differences in symptom status across the latent classes

A comparison of symptoms across the latent classes is reported in Table 3. The T-scores for anxiety, depression, fatigue, sleep disturbance, and pain interference were relatively lower in the sample according to the score manual of PROMIS-57 compared with the general population. There were significant differences in the mean T-scores for each domain among the latent classes, with the highest scores observed in Class 3 and the lowest scores observed in Class 1 ($P<0.05$).

Table 3 Symptoms across the latent classes (mean±standard deviation)

Domain	Class 1 (n=79)	Class 2 (n=74)	Class 3 (n=63)	*P* value

Anxiety	44.0±8.0	56.9±4.8	64.3±6.7	<0.001
Depression	43.9±7.3	56.5±4.5	62.7±6.4	<0.001
Fatigue	43.1±7.2	51.5±3.7	61.2±5.6	<0.001
Sleep disturbance	50.6±6.8	51.0±3.8	55.4±3.7	<0.001
Pain interference	48.0±9.5	57.5±5.5	64.7±5.2	<0.001

Demographic and clinical characteristics across the latent classes

Demographic and clinical characteristics of the latent classes are described in Table 4. The results of the chi-squared tests indicated that the proportion of patients assigned to the identified latent classes varied in terms of monthly family income (*P*=0.001); use of painkillers, antidepressants, or hypnotic drugs (*P*<0.001); MM bone disease (*P*<0.001); anemia (*P*=0.014); other complications (*P*=0.017); and daily step counts (*P*=0.043). ANOVA results indicated that there were no significant differences in daily sleep time (*P*=0.014) among the identified latent classes. No significant differences were identified for other variables.

Based on the outcomes of univariate analysis, a multinomial logistic regression analysis demonstrated that patients with low monthly family income (OR=3.14, *P*=0.010) and complications of MM bone disease (OR=2.95, *P*=0.029) were more likely to belong to Class 2. Additionally, patients treated with analgesics, antidepressants, or hypnotic drugs (OR=3.68, *P*=0.012) and those with daily step counts <5000 steps (OR=2.52, *P*=0.039) had a higher predisposition toward Class 3, which was also characterized by a high incidence of symptoms. Adequate daily sleep time (OR=0.74, *P*=0.031) played a protective role in avoiding a high incidence of symptoms (Table 5).

Table 4 Comparisons of demographic and clinical characteristics among the latent classes

**Variable**	**Class 1 (n=79)**	**Class 2 (n=74)**	**Class 3 (n=63)**	***P* value**
**Age (mean±standard deviation), years**	58.7±10.4	61.5±9.5	60.8±11.4	0.239
**Sex**				0.258
Male	35 (44.3%)	32 (43.2%)	20 (31.7%)	
Female	44 (55.7%)	42 (56.8%)	43 (68.3%)	
**Marital status**				0.320

For peer review only - http://bmjopen.bmj.com/site/about/guidelines.xhtml

Married	8 (10.1%)	3 (4.1%)	6 (9.5%)	
Separated/divorced/widowed	71 (89.9%)	71 (95.9%)	57 (90.5%)	
**Educational background**				0.477
Junior high school or below	53 (67.1%)	54 (73.0%)	46 (73.0%)	
High school	12 (15.2%)	14 (18.9%)	9 (14.3%)	
University or above	14 (17.7%)	6 (8.1%)	8 (12.7%)	
**Employment status**				0.814
Employed	6 (7.6%)	5 (6.8%)	3 (4.8%)	
Medical leave	2 (2.5%)	5 (6.8%)	3 (4.8%)	
Unemployed	25 (31.7%)	23 (31.1%)	20 (31.7%)	
Retired	28 (35.4%)	31 (41.8%)	23 (36.5%)	
Others	18 (22.8%)	10 (13.5%)	14 (22.2%)	
**Monthly family income (¥)**				0.001
<5000	50 (63.3%)	64 (86.5%)	52 (82.5%)	
≥5000	29 (36.7%)	10 (13.5%)	11 (17.5%)	
**MM type**				0.797
IgG type	42 (53.1%)	43 (58.1%)	34 (54.0%)	
IgA type	28 (35.4%)	27 (36.4%)	24 (38.1%)	
IgD type	4 (5.1%)	1 (1.4%)	4 (6.3%)	
Light chain type	4 (5.1%)	2 (2.7%)	1 (1.6%)	
Others	1 (1.3%)	1 (1.4%)	0 (0.0%)	
**Disease stage**				0.056
New diagnosis	6 (7.6%)	2 (2.7%)	5 (7.9%)	
Induction chemotherapy	34 (43.1%)	31 (41.9%)	26 (41.3%)	
Consolidation chemotherapy	22 (27.8%)	25 (33.8%)	15 (23.8%)	
Platform stage	14 (17.7%)	7 (9.4%)	6 (9.5%)	
Unclear	2 (2.5%)	4 (5.4%)	1 (1.6%)	
Recurrence	1 (1.3%)	5 (6.8%)	10 (15.9%)	
**Use of painkillers, antidepressants, or hypnotic drugs**				<0.001
No	71 (89.9%)	59 (79.7%)	39 (61.9%)	
Yes	8 (10.1%)	15 (20.3%)	24 (38.1%)	
**Complications**				
No	2 (2.5%)	1 (1.4%)	2 (3.2%)	0.860
Multiple myeloma bone disease	20 (25.3%)	41 (55.4%)	36 (57.1%)	<0.001
Impairment of renal function	9 (11.4%)	9 (12.2%)	14 (22.2%)	0.143
Anemia	17 (21.5%)	26 (35.1%)	28 (44.4%)	0.014
Hypercalcemia	2 (2.5%)	0 (0.0%)	3 (4.8%)	0.197
Thrombosis	1 (1.3%)	3 (4.1%)	1 (1.6%)	0.531
Inflammation	16 (20.3%)	16 (21.6%)	8 (12.7%)	0.355
Other complications	28 (35.4%)	13 (17.6%)	12 (19.0%)	0.017
**Treatments**				0.804
Oral drug therapy	3 (3.8%)	4 (5.4%)	1 (1.6%)	
Chemotherapy	64 (81.0%)	55 (74.3%)	51 (81.0%)	

For peer review only

Autologous hematopoietic stem cell transplantation	5 (6.3%)	9 (12.2%)	5 (7.9%)	
Others	7 (8.9%)	6 (8.1%)	6 (9.5%)	
**BMI**				0.198
<18.5	6 (7.6%)	2 (2.7%)	6 (9.5%)	
18.5–23.9	50 (63.3%)	52 (70.3%)	34 (54.0%)	
24–27.9	20 (25.3%)	13 (17.6%)	19 (30.2%)	
>27.9	3 (3.8%)	7 (9.5%)	4 (6.3%)	
**Daily sleep time (mean ± SD), hours**	7.2±1.4	6.7±1.4	6.5±1.5	0.014
**Daily step counts**				0.043
<5000	49 (62.0%)	54 (73.0%)	51 (81.0%)	
≥5000	30 (38.0%)	20 (27.0%)	12 (19.0%)	

Table 5 Results of multinomial logistic regression of latent class membership

**Characteristics**	**Class 2 (n=74)**		***P* value**	**Class 3 (n=63)**		***P* value**
	**OR**	**95% CI**		**OR**	**95% CI**	
**Monthly family income**						
≥5000	Ref			Ref		
<5000	3.14	1.32–7.44	0.010	2.33	0.95–5.70	0.064
**Use of painkillers, antidepressants or hypnotic drugs**						
No	Ref			Ref		
Yes	1.66	0.59–4.68	0.338	3.68	1.34–10.11	0.012
**Complication (multiple myeloma bone disease)**						
No	Ref			Ref		
Yes	2.95	1.11–7.83	0.029	2.81	0.98–8.07	0.055
**Anemia**						
No	Ref			Ref		
Yes	1.52	0.58–4.00	0.398	2.10	0.75–5.86	0.158
**Other complications**						
No	Ref			Ref		
Yes	0.75	0.28–2.00	0.563	1.04	10.36–3.00	0.946
**Daily sleep time**	0.83	0.64–1.07	0.143	0.74	0.57–0.97	0.031
**Daily step counts**						
≥5000	Ref			Ref		
<5000	1.59	0.74–3.42	0.234	2.52	1.05–6.05	0.039

OR, odds ratio; 95% CI, 95% confidence interval of OR

Between-group differences in functional status across the latent classes

For peer review only

A comparison of functional status across the latent classes is reported in Table 6 and Supplementary Figure 2. The T-scores for physical function, ability to participate in social roles and activities, and cognitive function were relatively lower in the sample according to the score manual of PROMIS-57, in which physical function was slightly impaired, compared with the general population. Significant differences were identified among the three classes, with the highest scores in all functional dimensions observed in Class 1 and the lowest scores observed in Class 3 (*P*<0.05).

Table 6 Functional status across the identified latent classes (mean±standard deviation)

**Domain**	**Class 1 (n=79)**	**Class 2 (n=74)**	**Class 3 (n=63)**	***P* value**
Physical function	50.2±6.7	44.0±4.1	37.8±7.9	<0.001
Ability to participate in social roles and activities	56.0±8.0	49.0±4.1	41.6±6.0	<0.001
Cognitive function	50.5±8.6	44.8±5.5	39.0±5.2	<0.001

Discussion

To the best of our knowledge, this is the first study to describe the homogeneous symptoms and associated factors in patients with MM using routinely captured PROs. Our findings revealed that a high prevalence of burdensome symptoms was found in this population during treatment, and 63.4% of patients reported different degrees of symptom burden, which deserves clinical attention.

The current study identified the target population with the most severe symptoms. Classes 2 and 3 represented groups with greater symptom burden, with patients in Class 3 being at a higher risk of symptom burden than those in Class 2. Depression, fatigue, and pain were identified as the most common symptoms in this study, consistent with the broader literature [14,15]. Boland et al. [14] reported that fatigue and bone pain were the most common symptoms in patients undergoing treatment for MM. A study on patients with stable but advanced-stage MM also confirmed that fatigue and pain were the most frequent symptoms [15]. Furthermore, Naegele et al. [16] reported that fatigue, diarrhea, and decreased appetite were the most frequent and distressing physical symptoms in patients with MM treated with autologous stem cell transplantation, and these

For peer review only

symptoms differed substantially before and after treatment. This discrepancy may be caused by differences in the treatment of patients. A systematic review identified pain, fatigue, sleep issues, and depression as the most frequently investigated symptoms in patients with MM [17]. Therefore, this study examined the association between demographic and cancer-related factors associated with different symptom profiles in patients with MM.

The demographic and cancer-related factors associated with different symptom profiles were identified in this study. An increase in age was not associated with a significant between-group difference. The median age in this sample was 60.3 years, which was relatively younger than the average age of patients with MM (73 years). Therefore, future studies should validate the results of this study in older patients. Complications of MM bone disease were predictive factors for high anxiety, depression, and pain interference. These findings are consistent with those of a previous study by Ebraheem et al. [18], suggesting that increased comorbidities and myeloma-related bone and kidney damage were associated with a higher rate of moderate to severe symptoms. Furthermore, depressive symptoms were found to increase the incidence of a higher number of comorbidities in patients with MM aged ≥65 years [19].

Consistent with previous reports, this study found that poor socioeconomic status was associated with increased symptoms of pain, suggesting that these patients may require additional support [20]. Low monthly family income was correlated with severe symptoms, particularly in the anxiety, depression, and pain domains. Lower income and unemployment have been reported as risk factors for financial burden as a direct result of cancer and related treatment and can be referred to as financial toxicity [20]. Previous studies have demonstrated that patients with MM have substantial areas of concern about the financial burden of disease (44%) and inability to perform daily activities (33%) and felt unhappy (41%). In addition, 36% reported pain and 15% reported depression [21], highlighting the need for multidisciplinary psychosocial support. According to Ramsenthaler et al., several patients with MM were burdened by financial worries, almost 50% of patients with MM reported concerns about dying, and almost 80% had financial

For peer review only

difficulties, of which 43.3% were severe [17]. Psychosocial stressors, including depression, cause high resistance to cell death, compromised immunity, and inflammatory and oxidative stress, which may affect cancer progression [22]. Additionally, these symptoms are associated with longer hospitalization, low medication adherence, and suicide [22]. Therefore, patients with poor socioeconomic status deserve more attention, particularly those with psychosocial symptoms. The use of painkillers, antidepressants, or hypnotic drugs and low physical activity were significant predictors of a high symptom burden in patients with MM. Patients using these drugs are more likely to experience severe pain, depression, and sleep issues and require medical attention than those who are not using these drugs.

Consistent with the results of previous studies, patients with greater symptom burden were more likely to report functional limitation than patients without burdensome symptoms [15,17,23]. For example, symptoms such as fatigue and pain have a negative effect on physical function and may cause treatment discontinuation [15,23]. In our study, patient physical function was slightly impaired compared with that of the general population, and significant differences were identified among the three classes. These results were consistent with those reported by Ramsenthaler et al. [17], suggesting that severely decreased social, physical, and cognitive functions were common among patients with MM. However, the class with lesser symptom burden was more likely to report high function levels, which may be due to selection bias. Differences in age may have contributed to these disparities, since relatively younger patients were included in this study. According to a national report in China, the mean age of patients diagnosed with MM was 58 years, which was approximately 10 years younger than that of Caucasians [24]. Since relatively young patients were included in this study, future studies should validate whether the findings can be generalized to older MM patient populations. Age is a well-established risk factor for cognitive decline, and impaired cognitive function is common in older patients. Older patients have been

For peer review only

more vulnerable to cognitive side effects of cancer treatments than younger patients [25]. Support for an interaction of age, cognitive reserve, and exposure to chemotherapy have been identified as risk factors for cognitive decline [26]. Hsu et al. reported that mild anxiety may be associated with better cognitive performance, and higher anxiety may be associated with poorer cognitive performance [27]. In a longitudinal prospective study of older patients with breast cancer, half of the investigated patients perceived a decline in cognitive function after 6 months of chemotherapy. However, there was no significant change in Mini-Mental Status Examination scores after chemotherapy or hormonal therapy over a short time in Janelsins' study [28]. There is a lack of study regarding the effects of cancer treatment on a cognitive performance in older patients with MM. Therefore, prospective, long-term studies are necessary to assess the effects of MM treatment on the cognitive function of older patients.

Limitations of the study

This study has some limitations. First, this was a cross-sectional study that did not explore the evolution of symptoms over time. Second, this study involved only two single centers, and the results need to be validated in multicenter studies. Moreover, the severity of the symptoms may have been underestimated, as some patients may have been significantly ill to participate in this study. Finally, this study did not evaluate treatment response. Further studies are required to incorporate more diverse sampling to fill ongoing knowledge gaps in this field.

Future Studies

In future studies, a longitudinal study design should be considered first to present the dynamic changes in patient symptom characteristics over time and over the course of disease. Second, multiple data collection methods (such as paper, Internet, or telephone collection) should be implemented to protect the integrity of the data. It is necessary to implement symptom

For peer review only

management based on PROs in heterogeneous populations.

Conclusion

Three distinct symptom profiles were observed in patients with MM during treatment. Patients with low monthly family income and complications of MM bone disease were more likely to report high levels of anxiety, depression, and pain interference than patients with high monthly family income and without complications of MM bone disease. Taking painkillers, antidepressants, or hypnotic drugs, with daily step counts <5000, and with functional limitations increased the risk of developing high symptom burden. The identification of patients with high symptom burden management needs should focus on assessing demographic and clinical characteristics and functional status so that interventions can be designed for high-risk individuals.

Acknowledgements

We thank the clinical staff at the study sites who enabled us to recruit the patients.

Contributors

CY1, CY2, and TC designed the study. CY1, NZ, GP, and WY were the principal investigators. TC and CY1 wrote the manuscript. CY1, TC, XL and TZ performed the statistical analyses, and all authors reviewed and contributed to the manuscript. All authors critically revised the manuscript and provided final approval.

Funding

This work was supported by the Science and Technology Project of Guilin City (20210227-8-5) and Project of the Guangxi Education Department (2021KY0521).

For peer review only

Competing interests

None declared.

Patient consent for publication

Not required.

Ethics approval

Ethical approval was obtained from the Institutional Review Board of the Second Affiliated Hospital of Guilin Medical University and all study sites (No. GLKJJH010).

Data availability statement

The data that support the findings of this study are available upon reasonable request from the corresponding author.

ORCID ID

Changrong Yuan, http://orcid.org/ 0000-001-8480-2569

REFERENCES

1 Rosenberg PS, Barker KA, Anderson WF. Future distribution of multiple myeloma in the United States by sex, age, and race/ethnicity. Blood 2015;125:410–2.

2 Kiely F, Cran A, Finnerty D, et al. Self-reported quality of life and symptom burden in ambulatory patients with multiple myeloma on disease-modifying treatment. Am J Hosp Palliat Care 2017;34:671–6.

For peer review only

3 Ramsenthaler C, Osborne TR, Gao W, et al. The impact of disease-related symptoms and palliative care concerns on health-related quality of life in multiple myeloma: a multi-centre study. BMC Cancer 2016;16:427.

4 Kamal M, Wang XS, Shi Q, et al. Symptom burden and its functional impact in patients with “symptomatic” relapsed or refractory multiple myeloma. Support Care Cancer 2021;29:467–75.

5 El-Jawahri A, Chen YB, Brazauskas R, et al. Impact of pre-transplant depression on outcomes of allogeneic and autologous hematopoietic stem cell transplantation. Cancer 2017;123:1828–38.

6 Cohen MZ, Rozmus CL, Mendoza TR, et al. Symptoms and quality of life in diverse patients undergoing hematopoietic stem cell transplantation. J Pain Symptom Manage 2012;44:168–80.

7 Sparano F, Cavo M, Niscola P, et al. Patient-reported outcomes in relapsed/refractory multiple myeloma: a systematic review. Support Care Cancer 2018;26:2075–90.

8 Rahman AF, Manatunga A, Guo Y, et al. A latent class analysis of PTSD symptoms among inner city primary care patients. J Psychiatr Res 2018;98:1–8.

9 Rimehaug SA, Kaat AJ, Nordvik JE, et al. Psychometric properties of the PROMIS-57 questionnaire, Norwegian version. Qual Life Res 2022;31:269–80.

10 Tang E, Ekundayo O, Peipert JD, et al. Validation of the Patient-Reported Outcomes Measurement Information System (PROMIS)-57 and -29 item short forms among kidney transplant recipients. Qual Life Res 2019;28:815–27.

11 Iverson GL, Marsh JM, Connors EJ, et al. Normative reference values, reliability, and item-level symptom endorsement for the PROMIS® v2.0 Cognitive Function-Short Forms 4a, 6a and 8a. Arch Clin Neuropsych 2021;7:1341–9.

12 Nylund KL, Asparouhov T, Muthén BO. Deciding on the number of classes in latent class analysis and growth mixture modeling: a Monte Carlo simulation study. Struct Equ Modeling

For peer review only

2007;14:535–69.

13 Watanabe M, Yamauchi K. Subtypes of overtime work and nurses' fatigue, mental status, and work engagement: a latent class analysis of Japanese hospital nurses. J Adv Nurs 2019;75:2122–32.

14 Boland E, Eiser C, Ezaydi Y, et al. Living with advanced but stable multiple myeloma: a study of the symptom burden and cumulative effects of disease and intensive (hematopoietic stem cell transplant-based) treatment on health-related quality of life. J Pain Symptom Manage 2013;46:671–80.

15 Jordan K, Proskorovsky I, Lewis P, et al. Effect of general symptom level, specific adverse events, treatment patterns, and patient characteristics on health-related quality of life in patients with multiple myeloma: results of a European, multicenter cohort study. Support Care Cancer 2014;22:417–26.

16 Naegele M, Kirsch M, Ihorst G, et al. Symptom experience of multiple myeloma (syMMex) patients treated with autologous stem cell transplantation following high-dose melphalan: a descriptive longitudinal study. Support Care Cancer 2017;26:833–41.

17 Ramsenthaler C, Kane P, Gao W, et al. Prevalence of symptoms in patients with multiple myeloma: a systematic review and meta-analysis. Eur J Haematol 2016;97:416–29.

18 Ebraheem MS, Seow H, Balitsky AK, et al. Trajectory of symptoms in patients undergoing autologous stem cell transplant for multiple myeloma: a population-based cohort study of patient-reported outcomes. Clin Lymphoma Myeloma Leuk 2021;21:e714–21.

19 Alobaidi A, Nabulsi NA, Talon B, et al. Depressive symptoms, mental health-related quality of life, and survival among older patients with multiple myeloma. Support Care Cancer 2020;28:4097–106.

For peer review only

20 Smith GL, Lopez-Olivo MA, Advani PG, et al. Financial burdens of cancer treatment: a systematic review of risk factors and outcomes. J Natl Compr Canc Netw 2019;17:1184–92.

21 Richter J, Sanchez L, Biran N, et al. Prevalence and survival impact of self-reported symptom and psychological distress among patients with multiple myeloma. Clin Lymphoma Myeloma Leuk 2021;21:e284–9.

22 Bortolato B, Hyphantis TN, Valpione S, et al. Depression in cancer: the many biobehavioral pathways driving tumor progression. Cancer Treat Rev 52:58–70.

23 Wang XS, Shi Q, Mendoza TR, et al. Minocycline for symptom reduction in patients with multiple myeloma during maintenance therapy: a phase II placebo-controlled randomized trial. Support Care Cancer 2021;29:6099–107.

24 Wang S, Xu L, Feng J, et al. Prevalence and incidence of multiple myeloma in urban area in China: a national population-based analysis. Front Oncol 2020;9:1513.

25 Ahles TA. Brain vulnerability to chemotherapy toxicities. Psychooncology 2012;21:1141–48.

26 Ahles TA, Saykin AJ, McDonald BC, et al. Longitudinal assessment of cognitive changes associated with adjuvant treatment for breast cancer: The impact of age and cognitive reserve. J Clin Oncol 2010;28:4434–40.

27 Hsu YH, Chen CH, Hsieh CC, et al. Subjective and objective cognitive functioning among patients with breast cancer: effects of chemotherapy and mood symptoms. Breast Cancer 2021;28:236–45.

28 Janelsins M C, Kohli S, Mohile S G, et al. An update on cancer- and chemotherapy-related cognitive dysfunction: current status. Semin Oncol 2011;38:431–38.

For peer review only

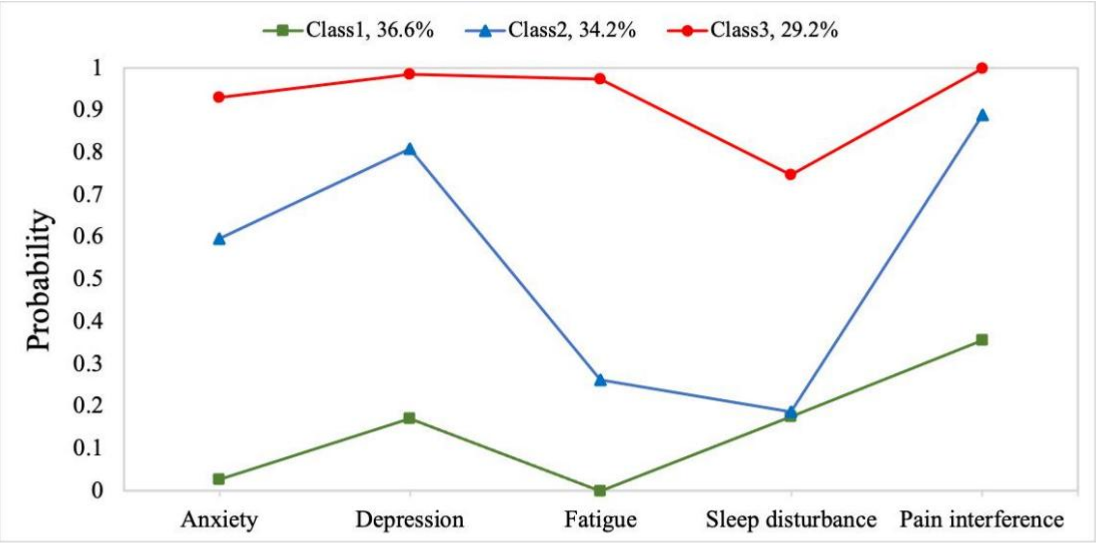

Supplementary Figure 1 The three-class latent profile analysis model

For peer review only

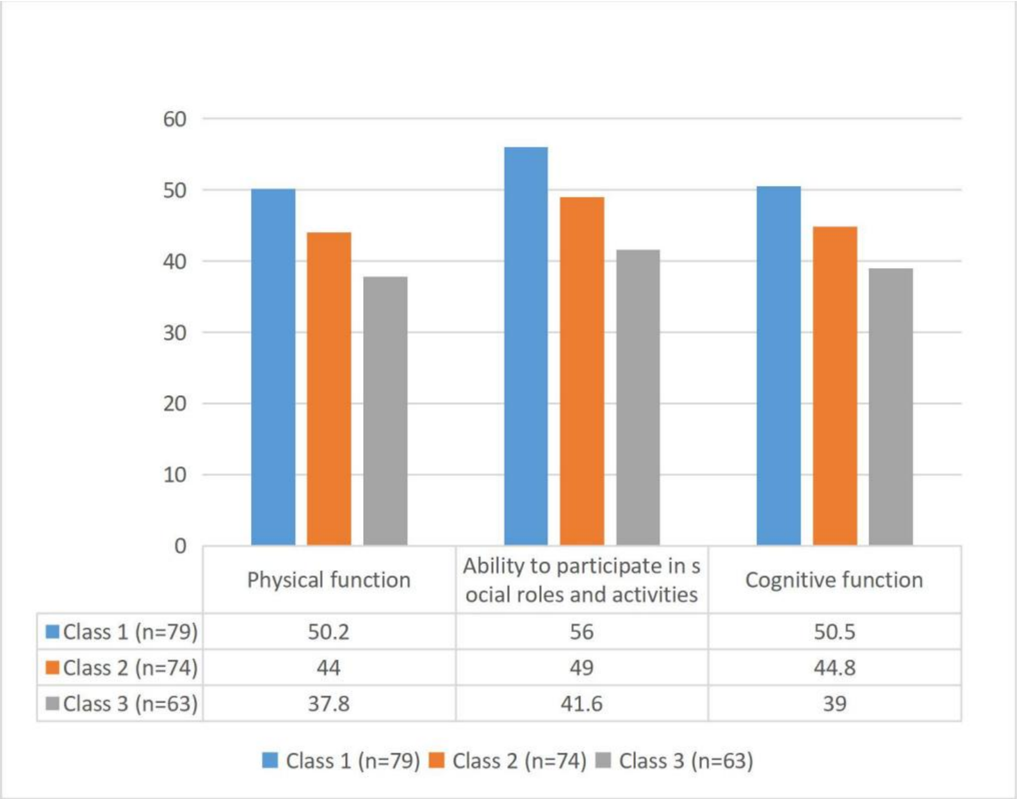

Supplementary Figure 2 Functional status across the identified latent classes

review only

Classification of symptom subtypes in patients with multiple myeloma during treatment: A cross-sectional survey study in China

Journal:	*BMJ Open*
Manuscript ID	bmjopen-2022-066467.R2
Article Type:	Original research
Date Submitted by the Author:	27-Dec-2022
Complete List of Authors:	Yu, Chunfang; The Second Affiliated Hospital of Guilin Medical University<br>Cai, Tingting; Fudan University School of Nursing<br>Zhou, Tingting; Fudan University School of Nursing<br>Zeng, Ning; Affiliated Hospital of Guilin Medical University<br>Liang, Xin; Guangxi Normal University<br>Pan, Guihua; Affiliated Hospital of Guilin Medical University<br>Ouyang, Wei; Peking Union Medical College Hospital, Department of Nursing<br>Yuan, Changrong; Fudan University School of Nursing,
**Primary Subject Heading**:	Nursing
Secondary Subject Heading:	Oncology, Research methods, Haematology (incl blood transfusion), Mental health
Keywords:	CHEMOTHERAPY, Lymphoma < HAEMATOLOGY, Myeloma < HAEMATOLOGY, Adult oncology < ONCOLOGY

BMJ

I, the Submitting Author has the right to grant and does grant on behalf of all authors of the Work (as defined in the below author licence), an exclusive licence and/or a non-exclusive licence for contributions from authors who are: i) UK Crown employees; ii) where BMJ has agreed a CC-BY licence shall apply, and/or iii) in accordance with the terms applicable for US Federal Government officers or employees acting as part of their official duties; on a worldwide, perpetual, irrevocable, royalty-free basis to BMJ Publishing Group Ltd ("BMJ") its licensees and where the relevant Journal is co-owned by BMJ to the co-owners of the Journal, to publish the Work in this journal and any other BMJ products and to exploit all rights, as set out in our licence.

The Submitting Author accepts and understands that any supply made under these terms is made by BMJ to the Submitting Author unless you are acting as an employee on behalf of your employer or a postgraduate student of an affiliated institution which is paying any applicable article publishing charge ("APC") for Open Access articles. Where the Submitting Author wishes to make the Work available on an Open Access basis (and intends to pay the relevant APC), the terms of reuse of such Open Access shall be governed by a Creative Commons licence – details of these licences and which Creative Commons licence will apply to this Work are set out in our licence referred to above.

Other than as permitted in any relevant BMJ Author's Self Archiving Policies, I confirm this Work has not been accepted for publication elsewhere, is not being considered for publication elsewhere and does not duplicate material already published. I confirm all authors consent to publication of this Work and authorise the granting of this licence.

For peer review only

Title Page

Manuscript category: research paper

Title: Classification of symptom subtypes in patients with multiple myeloma during treatment: A cross-sectional survey study in China

Authors and Affiliation:

Authors: Chunfang Yu[1#], MD; Tingting Cai[2#], PhD; Tingting Zhou[2], MD; Ning Zeng[3], MD; Xin Liang[4], PhD; Guihua Pan[3], RN; Wei Ouyang[5], RN; Changrong Yuan[2*], PhD

Note: Chunfang Yu and Tingting Cai made equal contributions to this manuscript and should be regarded as joint first author.

Affiliations:

[1]Department of Hematopathology, the Second Affiliated Hospital of Guilin Medical University, Guilin, China

[2]School of Nursing, Fudan University, Shanghai, China

[3]Affiliated Hospital of Guilin Medical University, Guilin, China

[4]School of Mathematics and Statistics, Guangxi Normal University, Guilin, China

[5]Department of Nursing, Peking Union Medical College Hospital, Beijing, China

Correspondence: Changrong Yuan, PhD, RN, FAAN, Fudan University, School of Nursing, 305 Fenglin Road Shanghai 200032, China (yuancr@fudan.edu.cn), Tel: 02164431803

Word counts: 3,573 words

For peer review only

Abstract

Objectives: To classify subgroups of cancer-related symptoms in patients with multiple myeloma (MM) during treatment and examine between-group differences in demographic and clinical characteristics in addition to functional status.

Design: Cross-sectional survey study

Setting: Hematology department of two tertiary hospitals affiliated with Guilin Medical University in China

Participants: Using a convenience sampling method, questionnaires were distributed to patients with MM visiting two hospitals in Guilin, China.

Interventions: The patients were categorized into subgroups based on cancer-related symptoms using a latent class analysis. An analysis of covariance was performed to examine how demographic and clinical characteristics and functional status differed among the subgroups.

Results: In total, 216 patients completed the survey, with an average age of 60.3 years. A three-class solution was identified: low symptom burden group (Class 1, 36.6%), moderate symptom burden group (Class 2, 34.2%), and high symptom burden group (Class 3, 29.2%). Patients with low monthly family income (odds ratio [OR]=3.14, P=0.010) and complications of MM bone disease (OR=2.95, P=0.029) were more likely to belong to Class 2. The predictors of high burden symptoms were treatment with painkillers, antidepressants, or hypnotic drugs (OR=3.68, P=0.012) and <5000 daily step counts (OR=2.52, P=0.039) in Class 3. Functional status was correlated with symptom burden, with patients in Classes 3 and 1 reporting significantly higher and lower functional status, respectively (P<0.05).

Conclusions: Patients with MM experienced varying degrees of symptoms during treatment. The

For peer review only

identification of patients with high symptom burden management should focus on the assessment of demographic and clinical characteristics, in addition to functional status.

Keywords: multiple myeloma, patient-reported outcomes, symptoms, function, latent class analysis

Strengths and limitations of this study

- A latent class analysis was used to categorize cancer-related symptoms in Chinese patients undergoing treatment for multiple myeloma.
- This study assessed between-group differences in the demographic and clinical characteristics and functional status of patients with multiple myeloma.
- The Patient-Reported Outcomes Information System (PROMIS)-57 and the PROMIS Cognitive Function Short Form were used as research tools.
- This study involved a limited number of treatment centers.
- Patient clinical characteristics did not include the evaluation of treatment response.

For peer review only

INTRODUCTION

Multiple myeloma (MM) is the second most common hematological cancer worldwide [1]. Although still incurable, advancements in the management of MM have extended survival, and MM is now considered a chronic disease. MM is primarily a cancer of old age with a median age at diagnosis of 73 years [1]. Older patients have a higher rate of comorbid chronic and progressive diseases than younger patients, and, compared to younger patients, a more careful approach is required to older patients during MM treatment to manage multiple treatment-related issues, including symptoms and functional limitations [2]. Patients with MM frequently report symptoms, including pain, fatigue, and depression, after diagnosis and throughout treatment [3]. With increasing survival rates, increased attention has focused on symptom control in this population.

Although advancements in MM treatment have been extensively reported, little is known about the symptoms of patients with MM during treatment [4-6]. Monitoring symptoms complements traditional laboratory testing and contributes to the identification of individuals who require supportive interventions. The effects of MM and its treatment may have an effect on patients' physical and emotional well-being, in addition to their social function. There is a paucity of data regarding the symptoms experienced by patients with MM, particularly those undergoing therapy. Symptoms may affect cancer treatment options and outcomes. Understanding individual symptom experiences is important to identify patients who are at risk of manifesting severe symptoms.

Evidence suggests that direct patient reports are the best way to capture information on patient experience of disease and related treatment [2, 6, 7]. Patient-reported outcomes (PROs) are strongly recommended for the measurement of symptoms and treatment effectiveness in patients with MM [7]. Specifically, among patients undergoing therapy, PROs can provide a better understanding of the magnitude and temporal trends of symptoms from the perspective of individual patients. Additionally, using PROs to capture patient symptoms is necessary to perform value-based and patient-centered care and to enable more accurate symptom evaluation [7].

To date, the heterogeneity of symptoms has not been captured among patients with MM.

For peer review only

Patients experience multiple symptoms that potentially interact with each other and that occur in clusters of clinically distinct subgroups. Statistical advancements in modeling contribute to the identification of subtypes and may be useful in distinguishing patients at risk. Latent class analysis (LCA) is a person-oriented approach used to classify individuals based on their symptom profiles and estimate distinct patterns [8].

There is no existing study to explore symptom profiles resulting from MM and its treatment in this population. Therefore, this study aimed to classify subgroups of patients with MM by incorporating PRO instruments and to examine between-group differences in demographic and clinical characteristics, in addition to functional status.

For peer review only

METHODS

Study design and sample

This study adopted a cross-sectional study design and adhered to the Strengthening The Reporting of OBservational Studies in Epidemiology guidelines. Convenience sampling was used to recruit patients with MM visiting the hematology department of two tertiary hospitals affiliated with Guilin Medical University in China between July and December 2021. Eligible patients were aged >18 years, diagnosed with primary MM, hospitalized for treatment, and able to speak and write Mandarin Chinese. Patients with other types of cancer or mental illnesses were excluded from this study.

Study instruments

Demographic and clinical data

Demographic and socioeconomic data included age, sex, marital status, educational background, employment status, monthly family income, medical insurance, daily sleep duration, and daily step counts. The MM type, disease stage, drug usage, complications, treatments, and body mass index (BMI) were retrieved from the medical records.

Patient-Reported Outcomes Measurement Information System (PROMIS)-57

Symptom experiences were measured using the Patient-Reported Outcomes Measurement Information System (PROMIS)-57 instrument. The PROMIS-57 consists of 57 items and includes five symptoms (anxiety, depression, fatigue, sleep disturbance, pain interference) and two functional domains (physical function, ability to participate in social roles and activities), along with one additional pain intensity item [9]. The instrument adopts a Likert scale (0–5) to assess the severity of symptoms or functional limitation, except for pain intensity, which has 11 response options (ranging from 0 to 10). The raw score of all outcome scores ranged from 4 to 20 and was converted into T-scores with a mean of 50 and a standard deviation (SD) of 10 in the scoring manual [10]. A higher score indicates poor functional status; however, for the symptom domain, this is reversed, with higher scores reflecting a high symptom burden [9]. Cronbach's α coefficient ranged from 0.85 to 0.95 in this study.

PROMIS-Cognitive Function Short Form

The four-item PROMIS-Cognitive Function Short Form measures assessed cognitive function over the preceding 7 days. Each question was given a response of 1 ("never") to 5 ("very often") [11]. The total raw scores ranged from 4 to 20 and were converted into T-scores. Similar to the PROMIS-57, lower T-scores indicate greater subjective cognitive difficulty. The Cronbach α for the scale was 0.93.

Data collection

The study protocol was approved by the Ethics Committee of the Second Affiliated Hospital of Guilin Medical University (No. GLKJJH010). Eligible patients were approached and provided with information about the study by assistant nurses. Written informed consent was obtained from all the participants. Both online and offline surveys were used to collect the data, and the assistant nurses assessed the questionnaires for completeness.

For peer review only

Statistical analyses

Statistical analyses were performed using the Statistical Package for the Social Sciences version 22.0 and Mplus version 8.0. Categorical and continuous variables are expressed as frequencies and proportions and medians with interquartile ranges, respectively. The models were run from one to six classes to determine the optimal number of latent classes. The model fit for LCA included the Akaike information criterion (AIC), Bayesian information criterion (BIC), sample size adjusted BIC (aBIC), entropy, and the Lo-Mendell-Rubin likelihood-ratio test (LMR-LRT). Lower AIC, BIC, and aBIC, higher entropy, and significant LMR-LRT indicate a better model fit [12,13].

After identifying the optimal latent class model, the symptom probabilities of identified subgroups were depicted by a line chart to present the characteristics of each group. To further identify between-group difference among the subgroups, an analysis of covariance was performed to examine how demographic, clinical characteristics, and functional status differed among the subgroups using the chi-squared and Fisher's exact tests. Subsequently, a multivariate logistic regression model was used to identify the factors associated with different classes, and the results were reported as adjusted odds ratios (ORs) with 95% confidence intervals (CIs). Additionally, a comparison of functional status was performed using one-way analysis of variance (ANOVA). Statistical significance was defined as $P<0.05$.

Patient and public involvement

During the design and preparation of the data collection tool, no study participants were involved in the development of the research question or the outcome measures.

RESULTS

Patient characteristics

The demographic and clinical characteristics of the patients are reported in Table 1. In total, 216 patients were available for the analysis, with an average age of 60.3 (SD=10.4 [range=22–84])

For peer review only

years. Most of the patients were female (59.7%), married (92.1%), and retired (38.0%), with an educational level of junior high school or below (70.8%) and a monthly family income below ¥5000 (76.9%). In terms of clinical characteristics, most patients had IgG MM (55.1%), were undergoing chemotherapy (70.8%), and had multiple concurrent complications of treatment (97.7%). Regarding treatment options, most patients underwent chemotherapy (78.7%) and did not require analgesia, antidepressants, or hypnotics before the diagnosis (97.7%). Most patients had a BMI within the normal range (63.0%) and reported daily step counts <5000 steps (71.3%).

Table 1 Demographic and clinical characteristics of the sample (n=216)

Variable	n (%)
**Sex**	
Male	87 (40.3)
Female	129 (59.7)
**Marital status**	
Married	199 (92.1)
Separated/divorced/widowed	17 (7.9)
**Educational background**	
Junior high school or below	153 (70.8)
High school	35 (16.2)
University or above	28 (13.0)
**Employment status**	
Employed	14 (6.5)
Medical leave	10 (4.6)
Unemployed	68 (31.5)
Retired	82 (38.0)
Others	42 (19.4)
**Monthly family income (¥)**	
<5000	166 (76.9)
≥5000	50 (23.1)
**MM type**	
IgG type	119 (55.1)
IgA type	79 (36.6)
IgD type	9 (4.2)
Light chain type	7 (3.2)
Others	2 (0.9)
**Disease staging**	
New diagnosis	13 (6.0)
Induction chemotherapy	91 (42.1)
Consolidation chemotherapy	62 (28.7)

For peer review only

Platform stage	27 (12.5)
Unclear	7 (3.2)
Recurrence	16 (7.4)
**Use of painkillers, antidepressants, or hypnotic drugs**	
No	169 (78.2)
Yes	47 (21.8)
**Number of complications**	
0	5 (2.3)
1	145 (67.1%)
2	44 (20.4%)
≥3	22 (10.2%)
**Types of complications**	
Multiple myeloma bone disease	97 (44.9)
Impairment of renal function	32 (14.8)
Anemia	71 (32.9)
Hypercalcemia	5 (2.3)
Thrombosis	5 (2.3)
Inflammation	40 (18.5)
Other complications	53 (24.5)
**Treatment**	
Oral drug therapy	8 (3.7)
Chemotherapy	170 (78.7)
Autologous hematopoietic stem cell transplantation	19 (8.8)
Others	19 (8.8)
**BMI**	
<18.5	14 (6.5)
18.5–23.9	136 (63.0)
24–27.9	52 (24.0)
>27.9	14 (6.5)
**Daily step counts**	
<5000	154 (71.3)
≥5000	62 (28.7)

Latent class analysis

The detailed results of the model fitting statistics for one to six classes are presented in Table 2. Among the types of model solutions, the three-class solution had the best fit (lowest AIC and BIC values), high entropy, significant *P* values for LMR and BLRT, and optimal clinical interpretability of each latent class. Therefore, the three-class solution was selected as the optimal solution.

For peer review only

Table 2 Model fit statistics for each of the fitted latent class analysis models

Model	AIC	BIC	aBIC	Entropy	LMR *P* value	BLRT *P* value	Classification probability
2C	1183.08	1220.21	1185.35	0.800	<0.001	<0.001	0.505/0.495
3C	1169.58	1226.96	1173.08	0.710	<0.001	<0.001	0.366/0.342/0.292
4C	1178.47	1256.10	1183.22	0.811	0.491	0.667	0.199/0.287/0.148/0.366
5C	1186.50	1284.38	1192.49	0.786	0.582	1.000	0.292/0.278/0.314/0.019/0.347
6C	1197.35	1315.49	1204.58	0.770	0.332	1.000	0.037/0.032/0.356/0.269/0.292/0.014

AIC, Akaike information criterion; BIC, Bayesian information criterion; aBIC, adjusted Bayesian information criterion; LMR, Lo-Mendell-Rubin; BLRT, bootstrap likelihood ratio test

For peer review only

Classification of symptoms and functional status

The item probabilities of the latent classes are shown in Supplementary Figure 1. Class 1 was characterized by low item probabilities for all symptom aspects and was labeled as the "low symptom burden group" (n=79, 36.6%). Class 2 was characterized by elevated item probabilities for anxiety, depression, and pain interference, whereas decreased item probabilities were associated with fatigue and sleep disturbance. Therefore, Class 2 was labeled as the "moderate symptom burden group" (n=74, 34.2%). Class 3 was characterized by high item probabilities for all symptoms and was labeled as the "high symptom burden group" (n=63, 29.2%).

Between-group differences in symptom status across the latent classes

A comparison of symptoms across the latent classes is reported in Table 3. The T-scores for anxiety, depression, fatigue, sleep disturbance, and pain interference were relatively lower in the sample according to the score manual of PROMIS-57 compared with the general population. There were significant differences in the mean T-scores for each domain among the latent classes, with the highest scores observed in Class 3 and the lowest scores observed in Class 1 (P<0.05).

Table 3 Symptoms across the latent classes (mean±standard deviation)

Domain	Class 1 (n=79)	Class 2 (n=74)	Class 3 (n=63)	*P* value

Anxiety	44.0±8.0	56.9±4.8	64.3±6.7	<0.001
Depression	43.9±7.3	56.5±4.5	62.7±6.4	<0.001
Fatigue	43.1±7.2	51.5±3.7	61.2±5.6	<0.001
Sleep disturbance	50.6±6.8	51.0±3.8	55.4±3.7	<0.001
Pain interference	48.0±9.5	57.5±5.5	64.7±5.2	<0.001

Demographic and clinical characteristics across the latent classes

Demographic and clinical characteristics of the latent classes are described in Table 4. The results of the chi-squared tests indicated that the proportion of patients assigned to the identified latent classes varied in terms of monthly family income (P=0.001); use of painkillers, antidepressants, or hypnotic drugs (P<0.001); MM bone disease (P<0.001); anemia (P=0.014); other complications (P=0.017); and daily step counts (P=0.043). ANOVA results indicated that there were no significant differences in daily sleep time (P=0.014) among the identified latent classes. No significant differences were identified for other variables.

Based on the outcomes of univariate analysis, a multinomial logistic regression analysis demonstrated that patients with low monthly family income (OR=3.14, P=0.010) and complications of MM bone disease (OR=2.95, P=0.029) were more likely to belong to Class 2. Additionally, patients treated with analgesics, antidepressants, or hypnotic drugs (OR=3.68, P=0.012) and those with daily step counts <5000 steps (OR=2.52, P=0.039) had a higher predisposition toward Class 3, which was also characterized by a high incidence of symptoms. Adequate daily sleep time (OR=0.74, P=0.031) played a protective role in avoiding a high incidence of symptoms (Table 5).

Table 4 Comparisons of demographic and clinical characteristics among the latent classes

**Variable**	**Class 1 (n=79)**	**Class 2 (n=74)**	**Class 3 (n=63)**	***P* value**
**Age (mean±standard deviation), years**	58.7±10.4	61.5±9.5	60.8±11.4	0.239
**Sex**				0.258
Male	35 (44.3%)	32 (43.2%)	20 (31.7%)	
Female	44 (55.7%)	42 (56.8%)	43 (68.3%)	
**Marital status**				0.320

For peer review only

Married	8 (10.1%)	3 (4.1%)	6 (9.5%)	
Separated/divorced/widowed	71 (89.9%)	71 (95.9%)	57 (90.5%)	
**Educational background**				0.477
Junior high school or below	53 (67.1%)	54 (73.0%)	46 (73.0%)	
High school	12 (15.2%)	14 (18.9%)	9 (14.3%)	
University or above	14 (17.7%)	6 (8.1%)	8 (12.7%)	
**Employment status**				0.814
Employed	6 (7.6%)	5 (6.8%)	3 (4.8%)	
Medical leave	2 (2.5%)	5 (6.8%)	3 (4.8%)	
Unemployed	25 (31.7%)	23 (31.1%)	20 (31.7%)	
Retired	28 (35.4%)	31 (41.8%)	23 (36.5%)	
Others	18 (22.8%)	10 (13.5%)	14 (22.2%)	
**Monthly family income (¥)**				0.001
<5000	50 (63.3%)	64 (86.5%)	52 (82.5%)	
≥5000	29 (36.7%)	10 (13.5%)	11 (17.5%)	
**MM type**				0.797
IgG type	42 (53.1%)	43 (58.1%)	34 (54.0%)	
IgA type	28 (35.4%)	27 (36.4%)	24 (38.1%)	
IgD type	4 (5.1%)	1 (1.4%)	4 (6.3%)	
Light chain type	4 (5.1%)	2 (2.7%)	1 (1.6%)	
Others	1 (1.3%)	1 (1.4%)	0 (0.0%)	
**Disease stage**				0.056
New diagnosis	6 (7.6%)	2 (2.7%)	5 (7.9%)	
Induction chemotherapy	34 (43.1%)	31 (41.9%)	26 (41.3%)	
Consolidation chemotherapy	22 (27.8%)	25 (33.8%)	15 (23.8%)	
Platform stage	14 (17.7%)	7 (9.4%)	6 (9.5%)	
Unclear	2 (2.5%)	4 (5.4%)	1 (1.6%)	
Recurrence	1 (1.3%)	5 (6.8%)	10 (15.9%)	
**Use of painkillers, antidepressants, or hypnotic drugs**				<0.001
No	71 (89.9%)	59 (79.7%)	39 (61.9%)	
Yes	8 (10.1%)	15 (20.3%)	24 (38.1%)	
**Complications**				
No	2 (2.5%)	1 (1.4%)	2 (3.2%)	0.860
Multiple myeloma bone disease	20 (25.3%)	41 (55.4%)	36 (57.1%)	<0.001
Impairment of renal function	9 (11.4%)	9 (12.2%)	14 (22.2%)	0.143
Anemia	17 (21.5%)	26 (35.1%)	28 (44.4%)	0.014
Hypercalcemia	2 (2.5%)	0 (0.0%)	3 (4.8%)	0.197
Thrombosis	1 (1.3%)	3 (4.1%)	1 (1.6%)	0.531
Inflammation	16 (20.3%)	16 (21.6%)	8 (12.7%)	0.355
Other complications	28 (35.4%)	13 (17.6%)	12 (19.0%)	0.017
**Treatments**				0.804
Oral drug therapy	3 (3.8%)	4 (5.4%)	1 (1.6%)	
Chemotherapy	64 (81.0%)	55 (74.3%)	51 (81.0%)	

For peer review only

Autologous hematopoietic stem cell transplantation	5 (6.3%)	9 (12.2%)	5 (7.9%)	
Others	7 (8.9%)	6 (8.1%)	6 (9.5%)	
**BMI**				0.198
<18.5	6 (7.6%)	2 (2.7%)	6 (9.5%)	
18.5–23.9	50 (63.3%)	52 (70.3%)	34 (54.0%)	
24–27.9	20 (25.3%)	13 (17.6%)	19 (30.2%)	
>27.9	3 (3.8%)	7 (9.5%)	4 (6.3%)	
**Daily sleep time (mean ± SD), hours**	7.2±1.4	6.7±1.4	6.5±1.5	0.014
**Daily step counts**				0.043
<5000	49 (62.0%)	54 (73.0%)	51 (81.0%)	
≥5000	30 (38.0%)	20 (27.0%)	12 (19.0%)	

Table 5 Results of multinomial logistic regression of latent class membership

**Characteristics**	**Class 2 (n=74)**		***P* value**	**Class 3 (n=63)**		***P* value**
	**OR**	**95% CI**		**OR**	**95% CI**	
**Monthly family income**						
≥5000	Ref			Ref		
<5000	3.14	1.32–7.44	0.010	2.33	0.95–5.70	0.064
**Use of painkillers, antidepressants or hypnotic drugs**						
No	Ref			Ref		
Yes	1.66	0.59–4.68	0.338	3.68	1.34–10.11	0.012
**Complication (multiple myeloma bone disease)**						
No	Ref			Ref		
Yes	2.95	1.11–7.83	0.029	2.81	0.98–8.07	0.055
**Anemia**						
No	Ref			Ref		
Yes	1.52	0.58–4.00	0.398	2.10	0.75–5.86	0.158
**Other complications**						
No	Ref			Ref		
Yes	0.75	0.28–2.00	0.563	1.04	10.36–3.00	0.946
**Daily sleep time**	0.83	0.64–1.07	0.143	0.74	0.57–0.97	0.031
**Daily step counts**						
≥5000	Ref			Ref		
<5000	1.59	0.74–3.42	0.234	2.52	1.05–6.05	0.039

OR, odds ratio; 95% CI, 95% confidence interval of OR

Between-group differences in functional status across the latent classes

For peer review only

A comparison of functional status across the latent classes is reported in Table 6 and Supplementary Figure 2. The T-scores for physical function, ability to participate in social roles and activities, and cognitive function were relatively lower in the sample according to the score manual of PROMIS-57, in which physical function was slightly impaired, compared with the general population. Significant differences were identified among the three classes, with the highest scores in all functional dimensions observed in Class 1 and the lowest scores observed in Class 3 (P<0.05).

Table 6 Functional status across the identified latent classes (mean±standard deviation)

**Domain**	**Class 1 (n=79)**	**Class 2 (n=74)**	**Class 3 (n=63)**	***P* value**
Physical function	50.2±6.7	44.0±4.1	37.8±7.9	<0.001
Ability to participate in social roles and activities	56.0±8.0	49.0±4.1	41.6±6.0	<0.001
Cognitive function	50.5±8.6	44.8±5.5	39.0±5.2	<0.001

Discussion

To the best of our knowledge, this is the first study to describe the homogeneous symptoms and associated factors in patients with MM using routinely captured PROs. Our findings revealed that a high prevalence of burdensome symptoms was found in this population during treatment, and 63.4% of patients reported different degrees of symptom burden, which deserves clinical attention.

The current study identified the target population with the most severe symptoms. Classes 2 and 3 represented groups with greater symptom burden, with patients in Class 3 being at a higher risk of symptom burden than those in Class 2. Depression, fatigue, and pain were identified as the most common symptoms in this study, consistent with the broader literature [14,15]. Boland et al. [14] reported that fatigue and bone pain were the most common symptoms in patients undergoing treatment for MM. A study on patients with stable but advanced-stage MM also confirmed that fatigue and pain were the most frequent symptoms [15]. Furthermore, Naegele et al. [16] reported that fatigue, diarrhea, and decreased appetite were the most frequent and distressing physical symptoms in patients with MM treated with autologous stem cell transplantation, and these

For peer review only

symptoms differed substantially before and after treatment. This discrepancy may be caused by differences in the treatment of patients. A systematic review identified pain, fatigue, sleep issues, and depression as the most frequently investigated symptoms in patients with MM [17]. Therefore, this study examined the association between demographic and cancer-related factors associated with different symptom profiles in patients with MM.

The demographic and cancer-related factors associated with different symptom profiles were identified in this study. An increase in age was not associated with a significant between-group difference. The median age in this sample was 60.3 years, which was relatively younger than the average age of patients with MM (73 years). Therefore, future studies should validate the results of this study in older patients. Complications of MM bone disease were predictive factors for high anxiety, depression, and pain interference. These findings are consistent with those of a previous study by Ebraheem et al. [18], suggesting that increased comorbidities and myeloma-related bone and kidney damage were associated with a higher rate of moderate to severe symptoms. Furthermore, depressive symptoms were found to increase the incidence of a higher number of comorbidities in patients with MM aged ≥65 years [19].

Consistent with previous reports, this study found that poor socioeconomic status was associated with increased symptoms of pain, suggesting that these patients may require additional support [20]. Low monthly family income was correlated with severe symptoms, particularly in the anxiety, depression, and pain domains. Lower income and unemployment have been reported as risk factors for financial burden as a direct result of cancer and related treatment and can be referred to as financial toxicity [20]. Previous studies have demonstrated that patients with MM have substantial areas of concern about the financial burden of disease (44%) and inability to perform daily activities (33%) and felt unhappy (41%). In addition, 36% reported pain and 15% reported depression [21], highlighting the need for multidisciplinary psychosocial support. According to Ramsenthaler et al., several patients with MM were burdened by financial worries, almost 50% of patients with MM reported concerns about dying, and almost 80% had financial

For peer review only

difficulties, of which 43.3% were severe [17]. Psychosocial stressors, including depression, cause high resistance to cell death, compromised immunity, and inflammatory and oxidative stress, which may affect cancer progression [22]. Additionally, these symptoms are associated with longer hospitalization, low medication adherence, and suicide [22]. Therefore, patients with poor socioeconomic status deserve more attention, particularly those with psychosocial symptoms. The use of painkillers, antidepressants, or hypnotic drugs and low physical activity were significant predictors of a high symptom burden in patients with MM. Patients using these drugs are more likely to experience severe pain, depression, and sleep issues and require medical attention than those who are not using these drugs.

Consistent with the results of previous studies, patients with greater symptom burden were more likely to report functional limitation than patients without burdensome symptoms [15,17,23]. For example, symptoms such as fatigue and pain have a negative effect on physical function and may cause treatment discontinuation [15,23]. In our study, patient physical function was slightly impaired compared with that of the general population, and significant differences were identified among the three classes. These results were consistent with those reported by Ramsenthaler et al. [17], suggesting that severely decreased social, physical, and cognitive functions were common among patients with MM. However, the class with lesser symptom burden was more likely to report high function levels, which may be due to selection bias. Differences in age may have contributed to these disparities, since relatively younger patients were included in this study. According to a national report in China, the mean age of patients diagnosed with MM was 58 years, which was approximately 10 years younger than that of Caucasians [24]. Since relatively young patients were included in this study, future studies should validate whether the findings can be generalized to older MM patient populations. Age is a well-established risk factor for cognitive decline, and impaired cognitive function is common in older patients. Older patients have been

For peer review only

more vulnerable to cognitive side effects of cancer treatments than younger patients [25]. Support for an interaction of age, cognitive reserve, and exposure to chemotherapy have been identified as risk factors for cognitive decline [26]. Hsu et al. reported that mild anxiety may be associated with better cognitive performance, and higher anxiety may be associated with poorer cognitive performance [27]. In a longitudinal prospective study of older patients with breast cancer, half of the investigated patients perceived a decline in cognitive function after 6 months of chemotherapy. However, there was no significant change in Mini-Mental Status Examination scores after chemotherapy or hormonal therapy over a short time in Janelsins' study [28]. There is a lack of study regarding the effects of cancer treatment on a cognitive performance in older patients with MM. Therefore, prospective, long-term studies are necessary to assess the effects of MM treatment on the cognitive function of older patients.

Limitations of the study

This study has some limitations. First, this was a cross-sectional study that did not explore the evolution of symptoms over time. Second, this study involved only two single centers, and the results need to be validated in multicenter studies. Moreover, the severity of the symptoms may have been underestimated, as some patients may have been significantly ill to participate in this study. Although the detail of anti-myeloma therapeutics could play an important role in the symptom experiences of MM patients, we did not specify the detail of chemotherapy. Finally, this study did not evaluate treatment response. Further studies are required to incorporate more diverse sampling to fill ongoing knowledge gaps in this field.

Future Studies

For peer review only

In future studies, a longitudinal study design should be considered first to present the dynamic changes in patient symptom characteristics over time and over the course of disease. Second, multiple data collection methods (such as paper, Internet, or telephone collection) should be implemented to protect the integrity of the data. It is necessary to implement symptom management based on PROs in heterogeneous populations.

Conclusion

Three distinct symptom profiles were observed in patients with MM during treatment. Patients with low monthly family income and complications of MM bone disease were more likely to report high levels of anxiety, depression, and pain interference than patients with high monthly family income and without complications of MM bone disease. Taking painkillers, antidepressants, or hypnotic drugs, with daily step counts <5000, and with functional limitations increased the risk of developing high symptom burden. The identification of patients with high symptom burden management needs should focus on assessing demographic and clinical characteristics and functional status so that interventions can be designed for high-risk individuals.

Acknowledgements

We thank the clinical staff at the study sites who enabled us to recruit the patients.

Contributors

CY1, CY2, and TC designed the study. CY1, NZ, GP, and WY were the principal investigators. TC and CY1 wrote the manuscript. CY1, TC, XL and TZ performed the statistical analyses, and all authors reviewed and contributed to the manuscript. All authors critically revised the manuscript and provided final approval.

For peer review only

Funding

This work was supported by the Science and Technology Project of Guilin City (20210227-8-5) and Project of the Guangxi Education Department (2021KY0521).

Competing interests

None declared.

Patient consent for publication

Not required.

Ethics approval

Ethical approval was obtained from the Institutional Review Board of the Second Affiliated Hospital of Guilin Medical University and all study sites (No. GLKJJH010).

Data availability statement

The data that support the findings of this study are available upon reasonable request from the corresponding author.

ORCID ID

Changrong Yuan, http://orcid.org/ 0000-001-8480-2569

REFERENCES

For peer review only

1 Rosenberg PS, Barker KA, Anderson WF. Future distribution of multiple myeloma in the United States by sex, age, and race/ethnicity. Blood 2015;125:410–2.

2 Kiely F, Cran A, Finnerty D, et al. Self-reported quality of life and symptom burden in ambulatory patients with multiple myeloma on disease-modifying treatment. Am J Hosp Palliat Care 2017;34:671–6.

3 Ramsenthaler C, Osborne TR, Gao W, et al. The impact of disease-related symptoms and palliative care concerns on health-related quality of life in multiple myeloma: a multi-centre study. BMC Cancer 2016;16:427.

4 Kamal M, Wang XS, Shi Q, et al. Symptom burden and its functional impact in patients with "symptomatic" relapsed or refractory multiple myeloma. Support Care Cancer 2021;29:467–75.

5 El-Jawahri A, Chen YB, Brazauskas R, et al. Impact of pre-transplant depression on outcomes of allogeneic and autologous hematopoietic stem cell transplantation. Cancer 2017;123:1828–38.

6 Cohen MZ, Rozmus CL, Mendoza TR, et al. Symptoms and quality of life in diverse patients undergoing hematopoietic stem cell transplantation. J Pain Symptom Manage 2012;44:168–80.

7 Sparano F, Cavo M, Niscola P, et al. Patient-reported outcomes in relapsed/refractory multiple myeloma: a systematic review. Support Care Cancer 2018;26:2075–90.

8 Rahman AF, Manatunga A, Guo Y, et al. A latent class analysis of PTSD symptoms among inner city primary care patients. J Psychiatr Res 2018;98:1–8.

9 Rimehaug SA, Kaat AJ, Nordvik JE, et al. Psychometric properties of the PROMIS-57 questionnaire, Norwegian version. Qual Life Res 2022;31:269–80.

10 Tang E, Ekundayo O, Peipert JD, et al. Validation of the Patient-Reported Outcomes Measurement Information System (PROMIS)-57 and -29 item short forms among kidney transplant recipients. Qual Life Res 2019;28:815–27.

For peer review only

11 Iverson GL, Marsh JM, Connors EJ, et al. Normative reference values, reliability, and item-level symptom endorsement for the PROMIS® v2.0 Cognitive Function-Short Forms 4a, 6a and 8a. Arch Clin Neuropsych 2021;7:1341–9.

12 Nylund KL, Asparouhov T, Muthén BO. Deciding on the number of classes in latent class analysis and growth mixture modeling: a Monte Carlo simulation study. Struct Equ Modeling 2007;14:535–69.

13 Watanabe M, Yamauchi K. Subtypes of overtime work and nurses' fatigue, mental status, and work engagement: a latent class analysis of Japanese hospital nurses. J Adv Nurs 2019;75:2122–32.

14 Boland E, Eiser C, Ezaydi Y, et al. Living with advanced but stable multiple myeloma: a study of the symptom burden and cumulative effects of disease and intensive (hematopoietic stem cell transplant-based) treatment on health-related quality of life. J Pain Symptom Manage 2013;46:671–80.

15 Jordan K, Proskorovsky I, Lewis P, et al. Effect of general symptom level, specific adverse events, treatment patterns, and patient characteristics on health-related quality of life in patients with multiple myeloma: results of a European, multicenter cohort study. Support Care Cancer 2014;22:417–26.

16 Naegele M, Kirsch M, Ihorst G, et al. Symptom experience of multiple myeloma (syMMex) patients treated with autologous stem cell transplantation following high-dose melphalan: a descriptive longitudinal study. Support Care Cancer 2017;26:833–41.

17 Ramsenthaler C, Kane P, Gao W, et al. Prevalence of symptoms in patients with multiple myeloma: a systematic review and meta-analysis. Eur J Haematol 2016;97:416–29.

18 Ebraheem MS, Seow H, Balitsky AK, et al. Trajectory of symptoms in patients undergoing

For peer review only

autologous stem cell transplant for multiple myeloma: a population-based cohort study of patient-reported outcomes. Clin Lymphoma Myeloma Leuk 2021;21:e714–21.

19 Alobaidi A, Nabulsi NA, Talon B, et al. Depressive symptoms, mental health-related quality of life, and survival among older patients with multiple myeloma. Support Care Cancer 2020;28:4097–106.

20 Smith GL, Lopez-Olivo MA, Advani PG, et al. Financial burdens of cancer treatment: a systematic review of risk factors and outcomes. J Natl Compr Canc Netw 2019;17:1184–92.

21 Richter J, Sanchez L, Biran N, et al. Prevalence and survival impact of self-reported symptom and psychological distress among patients with multiple myeloma. Clin Lymphoma Myeloma Leuk 2021;21:e284–9.

22 Bortolato B, Hyphantis TN, Valpione S, et al. Depression in cancer: the many biobehavioral pathways driving tumor progression. Cancer Treat Rev 52:58–70.

23 Wang XS, Shi Q, Mendoza TR, et al. Minocycline for symptom reduction in patients with multiple myeloma during maintenance therapy: a phase II placebo-controlled randomized trial. Support Care Cancer 2021;29:6099–107.

24 Wang S, Xu L, Feng J, et al. Prevalence and incidence of multiple myeloma in urban area in China: a national population-based analysis. Front Oncol 2020;9:1513.

25 Ahles TA. Brain vulnerability to chemotherapy toxicities. Psychooncology 2012;21:1141–48.

26 Ahles TA, Saykin AJ, McDonald BC, et al. Longitudinal assessment of cognitive changes associated with adjuvant treatment for breast cancer: The impact of age and cognitive reserve. J Clin Oncol 2010;28:4434–40.

27 Hsu YH, Chen CH, Hsieh CC, et al. Subjective and objective cognitive functioning among patients with breast cancer: effects of chemotherapy and mood symptoms. Breast Cancer

For peer review only

2021;28:236–45.

28 Janelsins M C, Kohli S, Mohile S G, et al. An update on cancer- and chemotherapy-related cognitive dysfunction: current status. Semin Oncol 2011;38:431–38.

For peer review only

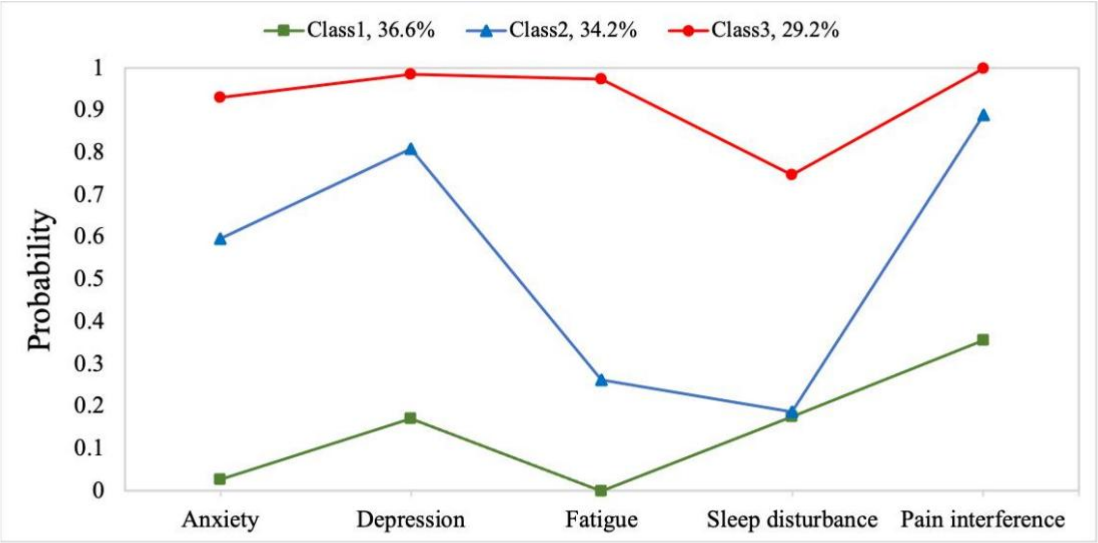

Supplementary Figure 1 The three-class latent profile analysis model

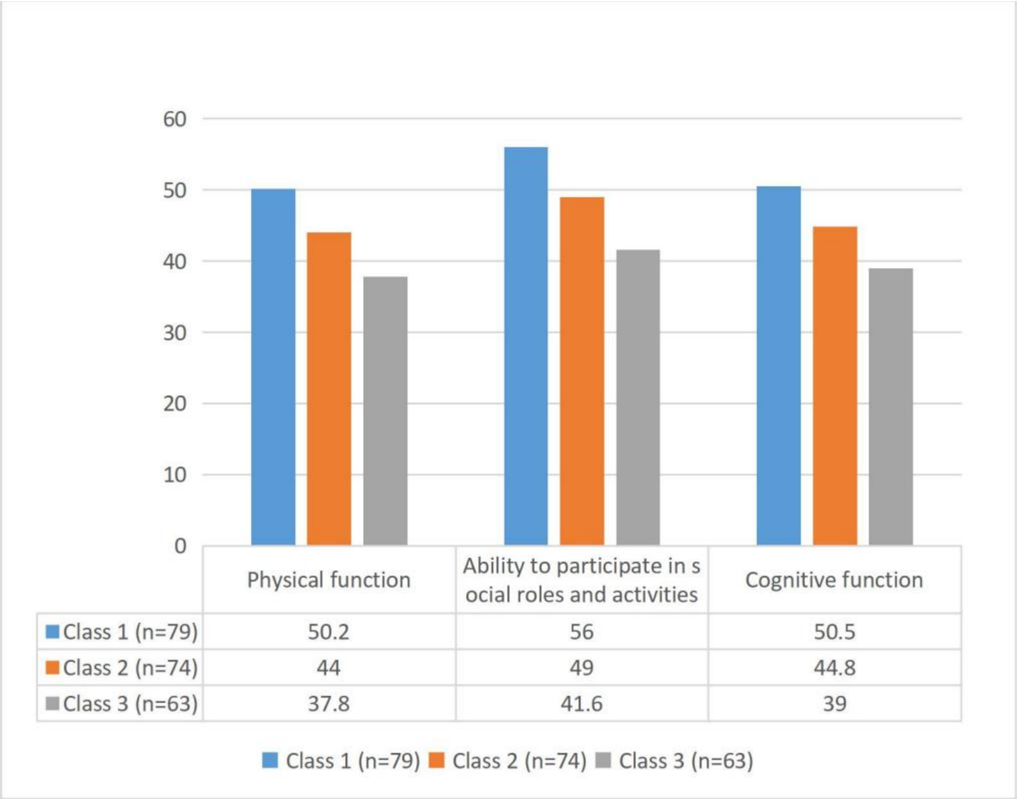

Supplementary Figure 2 Functional status across the identified latent classes

For peer review only
